# The Dawn and Advancement of the Knowledge of the Genetics of Migraine

**DOI:** 10.3390/jcm13092701

**Published:** 2024-05-04

**Authors:** Nader G. Zalaquett, Elio Salameh, Jonathan M. Kim, Elham Ghanbarian, Karen Tawk, Mehdi Abouzari

**Affiliations:** 1 Faculty of Medicine, American University of Beirut, Beirut 1107, Lebanon; 2 Department of Otolaryngology-Head and Neck Surgery, University of California, Irvine, CA 92697, USA; 3 Department of Neurology, University of California, Irvine, CA 92617, USA

**Keywords:** migraine, migraine with aura (MA), migraine without aura (MO), familial hemiplegic migraine (FHM), genetics

## Abstract

**Background:** Migraine is a prevalent episodic brain disorder known for recurrent attacks of unilateral headaches, accompanied by complaints of photophobia, phonophobia, nausea, and vomiting. Two main categories of migraine are migraine with aura (MA) and migraine without aura (MO). **Main body:** Early twin and population studies have shown a genetic basis for these disorders, and efforts have been invested since to discern the genes involved. Many techniques, including candidate-gene association studies, loci linkage studies, genome-wide association, and transcription studies, have been used for this goal. As a result, several genes were pinned with concurrent and conflicting data among studies. It is important to understand the evolution of techniques and their findings. **Conclusions:** This review provides a chronological understanding of the different techniques used from the dawn of migraine genetic investigations and the genes linked with the migraine subtypes.

## 1. Introduction

Migraine is a common episodic brain disorder known for its attacks of severe unilateral headaches, accompanied by photophobia, phonophobia, nausea, and vomiting [1,2,3]. According to the Global Burden of Disease Study in 2020, migraine remains second among the etiologies of disability [4,5], affecting 18% of women and 6% of men. Two prevalent types of migraine are migraine with aura (MA) and migraine without aura (MO). MA is a severe headache preceded by transient neurologic symptoms such as visual, sensory, and speech disturbances, which are not found in MO [6]. In addition, in the latest International Headache Society (IHS) criteria, MA includes motor and brainstem symptoms [1] (Table 1). The possible underlying mechanism of the aura is a brief wave of nervous system cell depolarization, propagating to the zones in the occipital lobe (cortical spreading depolarization), including the visual cortex, leading to the suppression of brain activity [7]. The exact relationship between cortical spreading depression (CSD) and headache is unknown, but there is evidence that CSD activates trigeminal nociceptors in rats [8,9].

Clinically, MA and MO are two different diagnosable entities, with the latter being more prevalent [10]. The international classification of headache disorder (ICHD-3) criteria for the diagnosis of the mentioned types of migraine are shown in Table 1 [11]. However, there is a historical unsettled debate on whether MO and MA are different disease entities or different manifestations of the same disease. This debate, while not directly related to the genetic basis of migraine, is an important aspect of the overall understanding of the condition and its subtypes.

**Table 1:** **a.** ICHD-3 criteria for migraine with aura diagnosis [11]. **b.** ICHD-3 criteria for migraine without aura diagnosis.

**a**	
A. At least 2 attacks fulfilling criteria B and C	
B. One or more of the following fully reversible aura symptoms:	
	1. Visual
	2. Sensory
	3. Speech and/or language
	4. Motor
	5. Brainstem
	6. Retinal
C. At least 2 of the following 4 characteristics:	
	1. At least 1 aura symptom spreads gradually over greater than or equal to 5 m, and/or more symptoms occur in succession
	2. Each individual aura symptom lasts 5–60 m
	3. At least 1 aura symptom is unilateral
	4. The aura is accompanied, or followed within 60 m, by a headache
D. Not better accounted for by another ICHD-3 diagnosis, and transient ischemic attack has been excluded	
**b**	
A. At least 5 attacks fulfilling criteria B–D	
B. Headache attacks lasting 4–72 h (when untreated or unsuccessfully treated)	
C. Headache has at 2 two of the following 4 characteristics:	
	1. Unilateral location
	2. Pulsating quality
	3. Moderate or severe pain intensity
	4. Aggravation by or causing avoidance of routine physical activity (e.g., walking or climbing stairs)
D. During headache at least one of the following occurs:	
	1. Nausea and/or vomiting
	2. Photophobia and phonophobia
E. Not better accounted for by another ICHD-3 diagnosis	

Note 1: When, for example, 3 symptoms occur during an aura, the acceptable maximal duration is 3 × 60 m. Motor symptoms may last up to 72 h. Aphasia is always regarded as a unilateral symptom, dysarthria may or may not be. Note 2: One or a few migraine attacks may be difficult to distinguish from symptomatic migraine-like attacks. Furthermore, the nature of a single or a few attacks may be difficult to understand. Therefore, at least five attacks are required. Individuals who otherwise meet the criteria for 1.1 Migraine without aura but have had fewer than five attacks should be coded 1.5.1 Probable migraine without aura. When the patient falls asleep during a migraine attack and wakes up without it, the duration of the attack is reckoned until the time of awakening. In children and adolescents (aged under 18 years), attacks may last 2–72 h (the evidence for untreated durations of less than two hours in children has not been substantiated).

Hemiplegic migraine, a debilitating chronic disorder diagnosed as familial (FHM) or sporadic (SHM), is a rare condition that comprises an aura and migraine stage. Affected individuals usually experience reversible neurological symptoms [12], such as hemiplegia or motor impairment, in the aura phase before the onset of migraine headaches [1,11]. The familial variant, an inherited autosomal dominant channelopathy [13], affects an individual’s first- or second-degree relatives [14], and can be divided into three unique types as follows [15]:

(1) FHM1 defined by mutations in the CACNA1A gene in chromosome 19,
(2) FHM2 with a mutant ATP1A2 gene in chromosome 1,
(3) FHM3 with SCN1A mutations in chromosome 2.

Although the genes implicated in the familial form are quite well understood [16,17], their role in conjunction with other unknown genes in the sporadic form is relatively obscure [18]. Sporadic hemiplegic migraine is akin to the familial version in that both share clinical commonalities and, in some cases, genetic causes [19]. To illustrate, a 57-year-old women, who displayed an array of symptoms, such as hemiparesis, had a genetic mutation (T1174s) in the sodium voltage-gated channel gene (SCN1A), which led to a sporadic hemiplegic migraine diagnosis; the aforementioned gene is also implicated in familial hemiplegic migraine, which suggests a genetic overlap between the two hemiplegic migraine variants [20]. Although many studies have found analogies between the two variants [21,22], the full extent of the genetic basis for the sporadic version remains contentious [23].

In this article, we aim to review the literature on the genetics of migraine. The goal of this review is to provide a chronological perspective on the advancements in the genetics of MO and MA since their first investigation. In addition, we aim to discuss the current knowledge of familial hemiplegic migraine.

## 2. Migraine without Aura and Migraine with Aura

**Figure 1:**
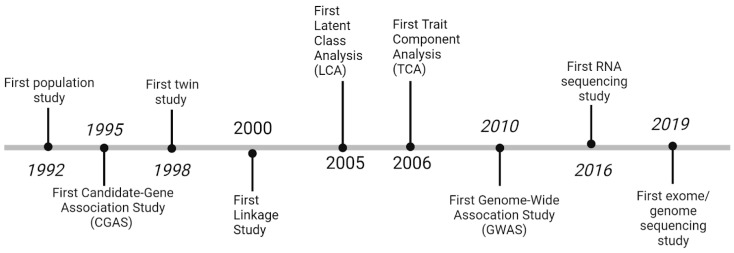
Chronology of techniques used to investigate migraine genetics.

The first population study on MO/MA genetics was published by Rasmussen et al. in 1992 [24], and the first twin study was published in 1998 by Ziegler et al. [25]. In 1995, the first candidate-gene association study (CGAS) was conducted by Frosst et al. [26]; however, the bulk of CGAS migraine research was published after the year 2004 [27,28,29,30,31,32,33,34,35,36]. Then, linkage studies, latent class analyses, and trait component analyses were adapted [37,38,39]. Finally, genome-wide association studies (GWAS), RNA sequencing, and exome/genome sequencing studies were applied to migraine genetics in 2010, 2016, and 2019 by Anttila et al. [40], Perry et al. [41], and Williams et al. [42], respectively. In this section, we will delve deeper into the findings of every research technique in migraine genetics. Figure 1 displays the chronology of MO/MA genetics research.

### 2.1. Genetic Load of MO and MA

Migraine has long been observed to cluster in families, with several patients having their first-degree relatives affected by the condition [43]. Starting in the 1990s, migraine and its genetic mechanism has been demonstrated by twin, family, and population studies [25,43,44,45,46,47,48]. Population-based studies have shown an increase in familial migraine risk [24,44,45,46,49]. Indeed, the risk of migraine was 50% higher in relatives of migraine probands [46]. Russel et al. showed that first-degree relatives of patients with MO had approximately a two-fold increased risk for MO, and 1.4 times the risk of having MA. In contrast, they showed that first-degree relatives of patients with MA had a four-fold increase in MA risk, but no increased risk for MO [44]. In addition, another study found a three-fold increase in the risk of MO and a two-fold increase in the risk of MA among first-degree relatives [24,43,49]. Additionally, twin studies provided a great insight into the heritability of migraine. For instance, Gervil et al. and Ulrich et al. analyzed Danish twin populations for the inheritance of MO and MA, respectively [48,50]. The results showed a higher pairwise concordance rate in monozygotic twins (MZ) when compared to dizygotic twins (DZ) (MA *p* < 0.001 and MO *p* < 0.05) (Table 2) [47,48,50,51,52]. In addition, pro-band-wise concordance was shown to be higher in MZ when compared to DZ in both MO and MA, as well as in different genders. Furthermore, a study of 30,000 twin pairs showed that genetic factors contribute equally to migraine phenotype as compared to the environment [53]. Finally, a recent study published in 2015 showed a heritability rate for migraine of 42% [54]. All of these published data lead to the conclusion that both MO and MA are a combination of genetics and environmental factors (e.g., stress and bright light) [54,55]. In addition, heritability was seen to be higher in migraine with aura than migraine without aura, leading to a higher genetic susceptibility [56,57,58].

Initially, due to the assumption that a migraine trait is a simple Mendelian transmission, several studies have been conducted, but have failed to clearly discern the mode of inheritance [59,60,61]. For instance, a study suggested a “sex-limited” inheritance of MO [62]. Another study suggested an autosomal recessive inheritance for MO and MA [60]. Several transmission patterns were hypothesized, but it is widely accepted nowadays that migraine is a genetic multifactorial trait [43,45,63]. Several genes have been correlated to MO and/or MA, which will be discussed below.

### 2.2. Are MO and MA Different Diseases?

Some clinicians might argue that MA and MO are different manifestations of the same disease [64,65]. Indeed, headache symptoms are virtually identical and might co-occur in the same patient [66], and the type of migraine can change over the years (aura attacks may develop in the elderly) [67]. The same prophylactic and treatment drugs are also effective in both [66]. However, each MO and MA has its own diagnostic criteria in the ICHD-3 [11], and genetic studies have shown different genetic loads for both [68,69].

Some studies have shown a common genetic basis for MA and MO. The international Brainstorm consortium, which compared genetic information between 265,218 patients and 784,643 controls, showed a significant genetic correlation between MO and MA [70]. In addition, the analysis of 23,000 single nucleotide polymorphisms (SNPs) showed that the majority of those analyzed were standard in MO and MA patients [71]; recently, Zhao et al. showed similar results by taking into account all available genetic information [72]. Conversely, several studies showed different genetic components for MO and MA [40,73]. Recently, a study analyzed the polygenic risk score of 21 migraine-associated SNPs and showed their association with MO only. However, many argue that research techniques such as genome-wide association studies (GWAS) particularly identify MO genes, as GWAS detects only top potential SNPs [2,3]. This study involved 152 MA patients compared to the 295 MO cases, which might lead to diminished statistical power when detecting MA genes [2]. In conclusion, with the available evidence, MO and MA are more alike than different; however, further studies are needed to discover the causal genes.

## 3. Various Techniques Unveiling the Genetic Basis of MO and MA

Several techniques have been used to characterize the genetic basis of MO and MA, starting with the population studies described above. The main methods used to reach this goal were as follows:

(1) candidate-gene association studies (CGAS),
(2) linkage studies,
(3) genome-wide association studies (GWAS),
(4) exome/genome sequencing,
(5) RNA and transcriptome sequencing.

### 3.1. Candidate-Gene Association Studies (CGAS)

For several years, the genetic basis of migraine was analyzed via focusing on hypothesized candidate genes from hypothesized migraine pathophysiological pathways. For instance, migraine has been linked to neurological, vascular, hormonal, and inflammatory pathways [74]. Using CGAS, approximately 100 genes were correlated with migraine [6].

Homocysteine is an excitatory amino acid that plays a role in the pathophysiology of cerebrovascular diseases [75]. Knowing that migraine has a cerebrovascular basis [27], researchers hypothesized that the genes responsible for homocysteine metabolism might be involved in the etiology of migraine. For example, the methylenetetrahydrofolate reductase gene (MTHFR), which is involved in the metabolism of folate, catalyzes the formation of 5-methylenetetrahydrofolate from 5,10-methylenetetrahydrofolate. The latter is the active form of folate and donates a carbon molecule for homocysteine for it to be converted into methionine [76]. A mutation in MTHFR was hypothesized to cause hyperhomocysteinemia and, consequently, migraine. Indeed, Frosst et al. reported an association between the homozygous C667T mutation of MTHFR and hyperhomocysteinemia [26]. Most studies identified the T-allele of the MTHFR C677T polymorphism to correlate with migraine, specifically MA (but no MO) [27,28,29,30,31,32,33,34,35,36]. Scher et al. studied 187 MA and 226 MO patients, in addition to 1212 control non-migraineurs. The group showed that the T/T MTHFR genotype was associated with increased odds of MA when compared to controls (odds ratio [OR], 2.05; 95% confidence interval; *p* < 0.006) [32]. Additionally, Lea et al. studied 652 Caucasian migraineurs and showed that the T/T genotype confers an increased risk for MA (OR: 2.0–2.5), but no increased risk for MO (*p* > 0.05) [29]. Conversely, a study by Todt et al. showed no association between the C667T genotype and MA (OR: 0.61–1.25 and *p* = 0.45) [77]. A possible explanation for their results was that their study’s sample sizes was composed of migraineurs with severe symptoms, and, thus, the MTHFR C667T allele could be found only in patients with mild to moderate MA [77]. Also, the International Headache Genetics Consortium (IGHC) data showed no clear evidence of MTHFR correlation in the 5175 migraineurs studied using genome-wide association studies (GWAS) [78]. 

The dopamine system has been hypothesized to be involved in the pathophysiology of migraine [79]. Studies have shown that D1 and D2 dopamine receptors exist in mice’s and rats’ trigeminal ganglion and trigeminal nucleus [80,81,82]. Additionally, studies have shown that administering apomorphine or piribedil (dopamine agonists) increases the cerebral blood flow [83,84]. Other animal studies have shown vasodilation in response to low dopamine doses and vasoconstriction with high doses [85]. As a result, researchers investigated the correlation between the genes involved in the dopaminergic pathway and migraine. The dopamine system is a series of steps, starting from phenylalanine and ending with norepinephrine and epinephrine [79]. Within these steps, dopamine is converted to norepinephrine by dopamine-β-hydroxylase (DBH), and norepinephrine is converted to epinephrine by catechol-O-methyltransferase (COMT) [79]. Finally, upon the release of dopamine in the synaptic cleft, a reuptake mechanism is mediated by presynaptic transporters called dopamine transporters (DAT1 and DAT2) [79]. As such, a mutation in any of the above genes would increase dopamine, and scientists hypothesized a potential increased migraine susceptibility. Two case–control studies have found an increased frequency of migraine in individuals with a homozygous COMT c.472 A > G (Val158Met) when compared to those with the Val/Val genotype [86,87]. However, Hagen et al. showed no association between the Val158Met polymorphism and migraine [88]. In addition, a study investigated the correlation of two SNPs, one within the promoter (−1021C→T) and another (+1603C→T) in exon 11 of the DBH gene in two different cohorts [89]. Results showed an association between the allelic and genotypic frequency distribution of DBH SNPs and migraine in both investigated cohorts [89].

Other genes of the serotonergic system, GABA-A receptor system, insulin receptors, estrogen receptors, LDL receptors, and ion transporters correlated with migraine due to their potential role in its pathophysiology and positive study results [66]. However, similarly to the case of MTHFR and COMT, most of the associations were not replicated and were subsequently disproven. For example, the study of 841 MA patients and 884 controls for thousands of genetic markers in 155 ion transport genes by Nyholt et al. was positive initially, but replication in an independent data set was negative [90]. In addition, 21 genes were associated with MA in another study, but the results could not be replicated in a larger data set [2,78]. Two other genes worth mentioning are the insulin receptor gene (INSR, chromosome 19p13) and the LDL receptor gene (19p13.2). These genes were associated with migraine, but were later disproven. The INSR gene was disproven in a sequencing study, and the LDL receptor gene was disproven because it could not be replicated in another study [91,92,93,94]. These disappointing results are due to small sample sizes (less than a few hundred cases), a lack of matching the samples for gender, age, and background, and diagnosis issues [2]. The lack of replication of most CGAS studies raises suspicion that other studies may be false positives; thus, other techniques were used to study the genetics of migraine.

### 3.2. Loci Linkage Studies

#### 3.2.1. Traditional Linkage Studies

Historically, linkage studies have contributed valuable inputs to the genetics of migraine by pinpointing chromosomal loci in families with migraine [66]. Initially, genotyping was achieved using microsatellite markers or genome-wide scans. For example, Russo et al. analyzed the genetics of 10 Italian families with MA and linked the loci 15q11-q13 with their MA diagnosis using regional microsatellite markers [95]. This locus represents the genomic region of three GABA-A receptor genes. Additionally, a study of a migraine family of 106 individuals from northern Sweden linked the 6p12.2-p21 locus with MO and MA through the use of genome-wide scanning [96]. Replication success for these linkage studies has been scarce, except for a few loci [66]. Wessman et al. and Bjornsson et al. pinned the 4q locus in studies involving Finnish and Icelandic families, respectively [97,98]. The Finnish study revealed locus 4q24 and the Icelandic study revealed locus 4q21 (Table 3). However, many unanswered questions remain concerning these loci; it is unclear whether they contain genes for MO, MA, or both. For these reasons, the validity of the traditional linkage studies results is questionable [66]. Other concerns include a high migraine prevalence and the subjective diagnosis of migraine, which can lead to difficulty in obtaining accurate pedigrees that can link migraine genes.

As a result, alternative linkage studies were used to eliminate this controversy, and two prominent alternatives were the latent class analysis (LCA) and trait component analysis (TCA). Using these methods, researchers can identify loci that could explain an underlying pathophysiological mechanism of a specific symptom [66].

#### 3.2.2. Latent Class Analysis (LCA)

Latent class analysis was introduced to eliminate the dichotomy of migraine diagnosis. This method focuses on multiple factors of migraine, including symptom severity, leading to a spectrum of clinical presentations. For example, Nyholt et al. [38] (frontrunners of LCA) and Ligthart et al. [104] clustered their patients based on migraine severity and associated symptoms. For instance, Nyholt et al. included pulsation in their classification, and classified their sample into four categories as follows: (1) asymptomatic individuals (CL0), (2) patients with a mild form of recurrent non-migrainous headaches (CL1), (3) patients with a moderately severe form of migraine, often without visual aura (CL2), and (4) patients with a severe form of migraine, often with aura (CL3) [38,64]. As expected, more individuals were labeled using the LCA approach, and none that were diagnosed using the IHS classification were missed [66]. Both of these studies pinned the 5q21 locus. The study by Ligthart et al. also reports the 10q22-q23 locus, in addition to another LCA study on the Australian and Finnish population [103]. This locus was reported using traditional linkage studies and TCA studies (Table 3).

#### 3.2.3. Trait Component Analysis (TCA)

As part of the effort to eliminate the diagnostic bias, researchers adopted the TCA method (starting with Palotie et al.) [103]. Similarly, TCA eliminates the dichotomous diagnostic approach of the IHS and uses the questionnaire information more optimally [103]. More specifically, researchers focus on specific trait components, or, in other words, individual clinical symptoms of migraine, and link chromosomal loci to this phenotypic group [39]. This could eliminate clinical heterogeneity and diagnostic issues. Loci 4q24, 17p13, and 10q22-q23 were linked to different migraine phenotypes using the TCA method (Table 4). Interestingly, 4q24 and 10q22-q23 were reported in Finnish and Australian linkage studies, respectively, using the IHS MA classification [97,103]. The latter mutation is the most significant, as it was replicated in Australian and Dutch studies [104]. The remaining gap unfilled by these new phenotyping methods is the identification of gene variants from the loci, which would give insight into the pathophysiology of specific symptoms and migraine in general.

### 3.3. Genome-Wide Association Studies

In the last decade, genome-wide association studies (GWAS) contributed significantly to our knowledge of the genetic basis of migraine. Unlike the other techniques, GWAS requires no prior hypothesis about the role of a DNA variant [105]. Instead, hundreds of thousands to millions of SNPs that are roughly equally dispersed in the genome are analyzed for association with a phenotype, and that is by comparing the results to the controls. The association is considered significant if the *p*-value is <5 × 10^−8^, according to the GWAS catalog [106]. This method has been effective in gene associations where other studies did not show results [105]. 

Ten migraine GWAS studies were conducted in the last decade, which were listed with their findings in Table 5 [40,63,73,74,107,108,109,110,111,112]. The first study was conducted by Anttila et al. in 2010 [40], and it consisted of 2748 patients with MA and 10,747 matched controls obtained from Finland, Germany, and the Netherlands. A single SNP reached genome-wide significance, which was the rs835740 on chromosome 8q22.1 (*p* = 5.38 × 10^−9^, OR = 1.23). This finding was replicated in a meta-analysis showing *p* = 1.69 × 10^−11^. This SNP is located between two genes implicated in glutamate homeostasis, which are MTDH (astrocyte elevated gene 1, AEG-1) and PGCP (plasma glutamate carboxypeptidase gene). MTDH has been shown to downregulate SLC1A2 (also known as GLT-1 and EAAT2) in cultured astrocytes; the latter gene encodes for the major glutamate transporters in the brain [113,114]. As such, a decrease in the activity of MTDH and/or PGCP (which metabolizes glutamate) will increase glutamate in the synaptic clefts. This was a plausible hypothesis for researchers as this neurotransmitter has been linked to the pathophysiology of migraine [40]. It is important to note that the relationship between MTDH and migraine remains controversial, as the correlation did not reach significance in subsequent studies [63,107]. Additionally, Gupta et al. [109] showed that the variant rs934937 on chromosome 6p24 increases the risk for migraine. This locus encodes for the PHACTR1 gene, which renders carriers susceptible to other vascular diseases, including coronary artery disease, cervical artery dissection, and hypertension. This gene was also suggested by Freilinger et al. [73] to correlate with MO. This gene was thought to affect the vascular system, and further studies have been completed to characterize its pathophysiological mechanism (check the fine mapping section below) [115]. 

Finally, the largest and most recent meta-analysis on migraine was conducted by Hautakangas et al. [112] in 2022, which included 102,084 migraine cases and 771,257 controls. The team identified three variants associated with MA as follows: (1) rs12598836 in HMOX2, (2) rs10405121 in CACNA1A, and (3) rs11031122 in MPPED2. HMOX2 is a constitutive gene that plays a role in heme catabolism, leading to antioxidant and anti-inflammatory effects [117]. CACNA1A encodes the alpha-1a subunit of the voltage-dependent P/Q calcium channel, and has been linked repeatedly to familial hemiplegic migraine (FHM), a subtype of MA [118]. Finally, MPPED2 is a metallophosphoesterase domain-containing protein which has been linked to various functions, including tumor suppression [119]. On the other hand, the meta-analysis suggested two variants associated with MO as follows: (1) rs7684253 in the locus near SPINK2, a serine peptidase inhibitor, and (2) rs8087942 in the locus near FECH, responsible for the synthesis of ferrochelatase.

At first, GWAS results seemed paradoxical, mainly because the results of these studies showed a more robust genetic association in MO [107], which is contradictory to the results from twin studies and population studies (showing that migraine with aura is more genetic). One possible explanation is that GWAS detects mainly variants with moderate or high allele frequencies (≥0.05); thus, relatively rarer alleles cannot be detected. Consequently, experts hypothesize that these rare alleles could be responsible for the genetic susceptibility of MA. As a result, researchers adopted RNA and exome/genome sequencing approaches to assess the contribution of such variants [3]. 

### 3.4. Fine Mapping of Potential Migraine Susceptible SNPs

Research was not limited to identifying possible SNPs using GWAS or other techniques. Instead, these potential loci were studied further using various methods. It is important to know that many of the SNPs correlated to migraine have unclear mechanisms of action. Thus, the fine mapping of these potential loci is of great value for understanding the genetics and pathophysiology of migraine. This approach occurs as follows: (1) association-test statistics are used to prioritize a set of SNPs that would likely contain disease-causing SNPs, (2) connecting these variants with genes using resources such as the Encyclopedia of DNA Elements (ENCODE), NIH Roadmap Epigenomics, and FANTOM5, and (3) conducting functional experiments to discern the exact pathophysiological mechanism of this variant/allele [6]. For example, the relationship of PHACTR1 to migraine has been investigated, and the pathophysiological mechanism has been suggested. After rs9349379 has been correlated to migraine (step 1), it was found to be on intron 3 of the PHACTR1 gene (step 2) [109]. Using the CRISPR-edited stem cell-derived endothelial cells, they demonstrated that this SNP regulates the endothelin 1 gene (EDN1), which is located 600 kb upstream of PHACTR1 and encodes a protein that promotes vasoconstriction, extracellular matrix production, fibrosis, and vascular smooth muscle cell proliferation (step 3) [120].

### 3.5. RNA Sequencing and Transcriptomic Studies

As discussed, GWAS detects high-frequency alleles exclusively, thus, rare variations that give insight into the genetics of migraine are not pinned by these studies. This problem was solved by using more specific techniques such as RNA sequencing and transcriptomic studies. To prevent the capturing bias, researchers have adopted RNA sequencing as a method to investigate migraine genetics. This technique allows investigators to identify novel transcripts, research the role of alternative splicing and gene fusion, and quantify the gene expression level related to migraine [121]. The final goal was also met using transcriptomic methods [41]. Table 6 summarizes studies in which RNA sequencing or transcriptomic studies were adapted. 

Renthal et al. (2018) [122] studied single-brain cell RNA sequencing data from cortical cells (neurons, oligodendrocytes, astrocytes, microglia, and endothelial cells). The analysis indicated that 70% and 30% of neuronal migraine-associated genes are significantly enriched in inhibitory and excitatory neurons, respectively, considering that many genes (such as SCN1A and CACNA1A) are found in both neuron types. Additionally, the study showed that 40% of known migraine-associated genes are enriched in a specific brain cell type. Vgontzas et al. (2020) [126] studied single-cell RNA sequencing data from the central and peripheral nervous system (neurons, glial cells, neurovascular cells). They showed that 11.1% of migraine-associated genes were selectively enriched in the central nervous system (HCK, ARHGEF26, WSCD1, TSPAN2, NEGR1, SLC24A3), 5.5% in neurovascular cells (i.e., GPR182, NOTCH4), and 3.7% in the peripheral nervous system (MYO1A, HELLS). Kogelman et al. performed RNA sequencing from the venous blood of MO and MA patients [125]. In 2019, the group compared 17 MO and 9 MA female patients to 20 female controls, and they showed that the genes NMNAT2 and RETN are differentially expressed in MA patients when compared to the controls; however, these results were not replicated in an independent cohort. In 2021 [127], the group compared the gene expression in MA and MO patients during the attack and after treatment. Results showed that 33 genes are differentially expressed between the two phases of migraine; most of these genes play a role in fatty acid oxidation (CPT1A, SLC25A20, and ETFDH), immune-related pathways (CARD9, SH2D2A, CD300C), and notch signaling pathways (MAML2, ADAM15, and ADAM17). Perry et al. [41] conducted a transcriptomic study of the expression of inflammation and immune response genes in chronic migraine patients’ calvarial periosteum. They found that 26 genes were upregulated and 11 genes were downregulated. The upregulated genes were associated with the activation of leukocytes, the production of cytokines, and the inhibition of NF-kB, while the downregulated genes were associated with the prevention of macrophage activation and cell lysis. The genes correlated to the pathophysiology of the periosteum are IL6, SOCS3, IFNB, CXCR4, CCL2, and NFKBIA.

### 3.6. Whole Exome or Whole Genome Sequencing (WES or WGS)

WES reveals nucleotide sequences in the coding region of the DNA, or the exon. WGS is more inclusive as it detects nucleotide sequences in both the coding and non-coding regions of the DNA (exons and introns). Applying the latter technique is important to identify the polymorphisms in the introns that might be responsible for migraine manifestation.

Ibrahim et al. completed whole exome sequencing on 16 individuals with no mutations in the FHM gene [128]. They associated ATP10A (p.Ala881Val) and ATP7B (p. Leu795Phe) variants with migraine. ATP10A encodes an ATPase with flippase activity on plasma membrane lipids, and ATP7B encodes transmembrane copper transporters. Interestingly, the ATP10A is found on locus 15q11-q13, which was pinned in 2005 by Russo et al. [95] using linkage studies (described previously). Additionally, the team suggested the possibility of CACNA1C (p.Ile662Leu) and CACNA11 (p.Arg111Gly) influence [128]. These genes encode voltage-gated calcium channels, similar to CACN1A1, which was pinned in FHM and MA (using GWAS). Another project detected the genes ATXN1 (contributes to glutamate signaling), FAM153B, and CACNA1B (voltage-gated calcium channels) in a population of 620 migraineurs [129,130]. This study was also replicated in 1930 migraine patients, and the same genes were detected. This work represents a combination of GWAS and RNA sequencing. However, it is important to mention that WES or WGS are expensive techniques that come with the burden of increased cost. They also impose some storage burden, which might affect the data quality [74]. In addition, these techniques might result in a capturing bias. For instance, WES is ineffective in capturing all mutations, particularly structural variants such as repetitive regions [131]. Also, migraine susceptibility loci are not limited to coding regions; many loci are in non-coding genomic regions that regulate splicing patterns or downstream genes [132]. Table 7 shows the genes hypothesized to be associated with migraine using WES/WGS.

### 3.7. Other Techniques

RT-PCR has been used on animal and cell models by Royal et al. [133] to study migraine genetics. The team studied two variants of the TRESK protein, a K+ channel encoded by the KCNK18 gene. These two variants are TRESK-MT and TRESK-C110R, which are non-functional variants of the potassium channel. Both were associated with migraine; however, only the TRESK-MT variant was shown to correlate with the MA phenotype, leading to the hyperexcitability of trigeminal neurons. The reason for this association is that TRESK-MT produces another variant, the TRESK-MT2, which co-assembles with TREK1 and TREK2, two other K+ channels, and inhibits them. Additionally, miRNA has been demonstrated to play a role in migraine pathophysiology [134,135]. miR-34a-5p and miR-382-5p have been shown to upregulate acutely during migraine attacks (both MO and MA); these markers were found in the blood and in cerebrospinal fluid (CSF), respectively [134]. Similarly, Tafuri et al. [135] showed that miRNA-27b was upregulated and miRNA-181a, miRNA-let-7b, and miRNA-22 were downregulated in MO patients when compared to healthy controls.

## 4. Monogenic Syndromes

The largest effect of migraine genetics was implied from rare monogenic syndromes with migraine symptoms. Such syndromes present as a set of symptoms, including migraine. As such, researchers correlated the genes mutated in those monogenic syndromes to migraine, which helped investigate the pathophysiological mechanism behind different types of migraine. Examples of these monogenic syndromes are included below.

### 4.1. CADASIL

Cerebral autosomal dominant arteriopathy with subcortical infarcts and leukoencephalopathy (CADASIL) is an inherited disease caused by a mutation in the *NOTCH3* gene found on chromosome 19. This gene encodes for a transmembrane receptor exclusively restricted to human vascular myocytes [136]. Histopathological studies of vascular tissue in CADASIL patients suggest the thickening and alteration of standard physiologic structure throughout the body [137]; however, the cerebral vasculature seems to be responsible for the majority of the disorder’s symptoms, usually including migraine, as the first presenting sign of the disease [136]. Interestingly, a study conducted by Tan et al. [138] showed that more than 75% of 300 symptomatic CADASIL patients experienced migraine, which were accompanied by auras approximately 90% of the time. However, other studies indicate different numbers. 

Nevertheless, taking all of the results together, migraine prevalence in CADASIL patients would be around 38%, which is still higher than the general population [138]. Several mechanisms have been proposed to explain the increased prevalence of migraine with auras in CADASIL patients as compared to the general population. One such mechanism centers around the idea that episodic ischemia generated by the vascular changes in the disease could be responsible for a more pronounced cerebral hypoperfusion phase, leading to cerebral blood flow changes similar to those observed in CSD [139], and thereby accounting for more severe auras [140]. Other plausible mechanisms include the possibility that the vascular abnormalities in CADASIL patients could decrease the threshold for CSD, as demonstrated in mice with mutated or deleted NOTCH3 genes [141], that the brainstem involvement in the disease process in CADASIL patients increases their susceptibility for migraine with auras, or that the NOTCH3 gene is involved in the pathway of migraine auras, since genetic studies have shown that family members of migraine patients have an increased risk of experiencing migraine themselves [44,142].

### 4.2. D-CAA

Cerebral amyloid angiopathy (CAA) is a cerebrovascular disease characterized by the accumulation of β-amyloid molecules in the leptomeninges of the central nervous system and the cerebral vessels [143]. This disease can lead to a severe intracerebral hemorrhage (ICH) in elderly patients [144]. However, preceding the ICH symptoms, migraine with aura often manifests as a presenting sign and an early marker of hereditary cases of CAA, especially Dutch-type CAA (D-CAA). This was seen in a study conducted by Koemans et al. [145], which found a 56% prevalence of migraine with aura in 86 recruited D-CAA patients. Interestingly, migraine was the initial symptom in approximately 80% of the cases [145]. As is the case with other cerebrovascular angiopathies, the exact mechanism behind the onset of migraine in this type of disease is not very well understood. However, several similar theories to the ones mentioned previously have also been suggested.

### 4.3. COL4A1-Related Disorders

COL4A1 is a gene located on chromosome 13 that encodes for the α-1 subunit of type IV collagen. This subunit plays an important role in the basement membrane of several different tissues in the body, especially the vascular tissue surrounding the blood vessels. Mutations of this gene cause a COL4A1-related brain small-vessel disease, which targets fragile vessels; this leads to hereditary infantile hemiparesis, retinal arteriolar tortuosity and leukoencephalopathy, and familial porencephaly [146,147]. Several studies show that migraine with aura may be a symptom of this mutation, as presented in a study of six affected family members, where 50% presented with auras [148]. This is also seen in a systematic review conducted by Lanfranconi et al. [149], in which 10 out of 52 carrier subjects had experienced migraine.

### 4.4. FASPS

Familial advanced sleep-phase syndrome (FASPS) is an autosomal dominant disorder caused by a missense mutation in the CSNK1D gene, which encodes for the Casein Kinase Iδ (CK1δ) [150], a serine/threonine kinase which phosphorylates several important target proteins in order to regulate the cell cycle, cell differentiation, proliferation, and the circadian clock [151,152]. Patients usually experience an earlier sleep onset and morning awakening, often described as “morning larks” [153]. Interestingly, in two different mutations (T44A and H46R) of the CSNK1D gene in transgenic mice, a co-segregation was also found with MA [150,154,155]. In essence, sensitization to pain resulting from nitroglycerin-triggered migraine reduced the threshold for CSD, and increased calcium signaling were detected in the T44A transgenic mice [150,155], thus explaining the co-presence of MA with the disease. Involved in migraine pathogenesis, the CSNK1D gene provides evidence for the involvement of the hypothalamus in the development of and susceptibility to migraine.

### 4.5. KCNK18

The TWIK-related spinal cord potassium channel (TRESK) is a member of the two-pore domain potassium (K2P) channel family—an important modulator of the resting membrane potential—encoded by the KCNK18 gene [156]. A frameshift mutation in this gene produces a truncated and non-functional channel, which can also suppress the levels of the wild-type channel and increase the susceptibility to migraine with aura [157]. This mutation was first discovered in a patient suffering from MA, and was later also confirmed in seven of the patient’s relatives who also suffered from the same disease [156].

### 4.6. ROSAH Syndrome

Heterozygous missense variants of the α-kinase gene ALPK1 are responsible for the pathogenesis of ROSAH syndrome, named after its five main symptoms: retinal dystrophy, optic nerve edema, splenomegaly, anhidrosis, and migraine headache [42]. This gene has been detected at high levels in the retina, in the retinal pigment epithelium, and in the optic nerve. It is important to note that migraine is also a frequent feature of the disease.

### 4.7. HERNS

Hereditary endotheliopathy with retinopathy, nephropathy, and stroke (HERNS) is an autosomal dominant systemic multi-infarct disorder that was first described by Jen et al. [158] in 1997 in a Chinese American family. As its name implies, this disease first manifests as visual impairment due to macular edema and as renal dysfunction with albuminuria [158]. The neurologic symptoms usually appear in the second decade of life, most commonly emerging as migraine headaches, in addition to psychiatric manifestations, hemiparesis, dysarthria, and others [158,159]. The mechanism behind the disease is generalized vascular damage in different capillaries and arterioles of the body, including retinal, cerebral, and renal areas [158,160]. 

### 4.8. MELAS

Mitochondrial encephalopathy, lactic acidosis, and stroke-like episodes (MELAS) syndromes are most commonly caused by an A to g transition mutation at position 3243 of the mitochondrial genome [161,162]. It is characterized by recurrent attacks of migraine-like headaches with vomiting, epilepsy, and stroke-like episodes, accompanied with blindness, deafness, cognitive impairment, and cardiac conduction defects, among others [163,164,165,166]. Even though the transition cited previously is the primary mutation seen in MELAS, it is, however, a polygenic disease caused by several mutations that involve mitochondrial tRNA and protein-coding genes, some of which are also involved in other mitochondrial diseases, such as LHON, Leigh Disease, and MERRF [167]. However, surprisingly, studies performed by Buzzi and colleagues [168] and Cevoli et al. [169] on maternal lineages with MELAS showed that most subjects were monosymptomatic, with the disease manifesting only as migraine. In addition, all of the migraine-only subjects did not carry the 3243 A > G tRNA Leu (MELAS) mutation, suggesting that this mutation does not contribute to the maternal multigenerational migraine with or without aura [168]. 

### 4.9. RVCL-S

Retinal vasculopathy with cerebral leukoencephalopathy and systemic manifestations (RVCL-S) is a rare systemic small-vessel disease caused by an autosomal dominant mutation in the three-prime repair exonuclease 1 (TREX1), mainly affecting the white matter of the CNS [170,171]. The amyloid-negative angiopathy involves mostly small vessels such as arterioles and capillaries in several locations of the body, including the retina and the brain [172]. This disorder is characterized by retinopathy, neurological deficits, and other systemic symptoms, including anemia, liver disease, kidney injury, and Raynaud’s phenomenon [170]. Migraine with and without aura are sometimes also reported by affected patients, as reported by 42% of patients in cross-sectional studies [172,173,174,175]. These kinds of migraine tend to occur in adult RVCL-S patients, compared to the earlier onset (childhood or adolescence) in the general population, which could suggest that vasculopathy is responsible for the onset of the migraine in these patients [175].

### 4.10. CCM

Familial cerebral cavernous malformations (CCM) is a heritable autosomal dominant disease characterized by at least three mutations in three different loci as follows: CCM1 on chromosome 7q, CCM2 on chromosome 7p, and CCM3 on chromosome 3p, characterized by vascular abnormalities in the central nervous system (CNS), leading to epileptic seizures and hemorrhagic strokes [176,177,178,179]. Several studies have also found migraine to be a symptom of this disorder [179].

## 5. Familial Hemiplegic Migraine (FHM)

**Figure 2:**
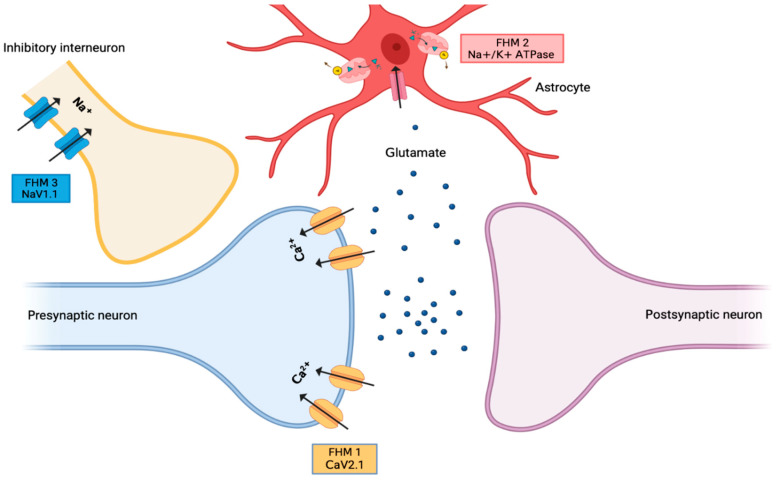
Figure representing the channels inhibited by each type of familial hemiplegic migraine.

As discussed, familial hemiplegic migraine (FHM) represents a rare autosomal dominant subtype of MA with an obligatory presence of a motor aura, represented by reversible motor weakness—hence the “hemiplegic” part of the disease—that is most often, but not always, unilateral [180,181]. Additionally, the diagnostic guidelines of the third edition of the International Classification of Headache Disorders, provided by the Headache Classification Committee of the International Headache Society, require the presence of at least one first- or second-degree relative having a migraine with motor auras (Table 8) [11]. The age interval of clinical appearances is flexible, stretching from 5 to 30 years old in most cases, with migraine tending to appear more in younger people [182]. Aside from the essential motor aura symptoms, a population-based study by Thomsen et al. showed that the other most common aura types were sensory, visual, and aphasia [183]. Even though motor, sensory, and visual auras were essentially similar to those seen in MA, their duration was significantly longer in FHM than in MA [180]. Many trigger factors have been implicated in the appearance of FHM, including acute stress, emotional fluctuation, excess or lack of sleep, minor head trauma, and menstruation in women [184,185,186]. In addition, more than two-thirds of FHM patients displayed a co-occurrence of basilar migraine (BM) as well, defined according to the IHS guidelines [183]. An overlap between epilepsy and migraine has also been suggested by the presence of seizures in certain specific pathogenic cases of FHM [187,188]. Being genetically heterogeneous, FHM has been divided into three subtypes, based on the genetic mutation responsible for the disease presentation (Figure 2).

### 5.1. FHM1

Familial hemiplegic migraine type 1 (FHM1) was first identified to be related to a specific genetic mutation in 1996, when Ophoff et al. demonstrated the presence of a CACNA1A mutation on chromosome 19p13. This gene encodes the pore-forming α1 subunit of the P/Q type calcium channel CaV2.1, which is found on presynaptic and somatodendritic membranes [21,189]. In fact, the study found four missense mutations associated with the presentation of the disease. However, several other mutations have been added to the list [190,191]. 

#### 5.1.1. Calcium Channels

As indicated by Bolay et al. [8], the most plausible and acceptable mechanism of migraine auras today is an increased cortical spreading depression (CSD) in the brain; genetic mutations in the aforementioned trio of genes are linked with augmented concentrations of neurotransmitters and potassium ions at the synaptic cleft, which may cause the cortical spreading depression commonly seen in migraine aura [192]. Contemporary studies that have physiologically induced visual auras have implicated cortical spreading depression in the onset of a migraine aura, which is accompanied by symptoms such as visual, language, or motor impairments [193]. Although the etiology of a migraine aura remained highly debated, understanding the involvement of specific channels may provide valuable insights. In recent studies involving mice, those with R192Q or S218L missense mutants in the α1 subunit of the Cav2.1 Ca2+ channels exhibited spontaneous cortical spreading depression events (CSD); mutant mice had a reduced threshold and a greater propagation speed for these events, which align with FHM1 clinical phenotypes [194].The role of CaV2.1 channel activity in CSD has been thoroughly investigated by Ayata et al. [195] using in vivo cortical microdialysis on leaner and tottering mice, with tg^la^ and tg mutations in the α1A subunit of CaV2.1, respectively. These mutations have been shown to decrease the density of Ca2+ currents significantly and increase the activation threshold of CaV2.1 channels, thereby reducing the probability of their activation when compared to wild-type mice [196]. In essence, the previously mentioned in vivo studies showed a two-fold reduction in glutamate release in the mutant mice as compared to the wild type and a 10-fold increase in the resistance to CSD following KCl-induction and electrical stimulation [195]. As such, these findings support the assumption that a decreased Ca2+ influx through the CaV2.1 channels increases the resistance to CSD, hence decreasing the plausibility of an aura. Therefore, it would be logical to assume that the mutations seen in FHM1 should have an opposite gain-of-function effect to increase the susceptibility of CSD in patients. 

#### 5.1.2. Specific Mutations

A study conducted by van den Maagdenberg et al. [197] on knockin transgenic mice models with the R192Q human mutation responsible for FHM1 found that CaV2.1 channels in the mutant mice open more rapidly and have a lower activation threshold, thereby opening at lower potentials when compared to wild-type channels. In addition, the current density through the mutant CaV2.1 channels was higher than that in wild-type channels, and neurotransmission at the synapses was also increased through an elevated neuromuscular junction concentration of glutamate with approximately constant concentrations of GABA, an inhibitory neurotransmitter [197]. Other studies also showed that the increased contribution of these P/Q calcium channels causes an increase in the release of glutamate by cortical neurons at physiologic microtubule Ca2+ levels [198]. These findings support the previously stated hypothesis that FHM1 results from gain-of-function mutations of the CaV2.1 channels, leading to a reduced threshold for the CSD. This was further supported by Eikermann-Haerter and colleagues [199], who showed that mutant mice with the same R192Q mutation had an elevated frequency of CSD and an increased speed of propagation following KCl induction stimulation studies. Even though R192Q mutant mice expressed pure FHM1 symptoms with hemiplegia only, S218L, another studied mutation in the same knockin mice, showed a more severe phenotype, characterized by seizures, cerebellar symptoms, coma, and possibly fatal cerebral edema occurring after minor head trauma due to more severe calcium channel dysfunction [197,199]. In addition, further studies showed that the underlying mechanism for the phenotypic differences between these two mutations is the level of the subcortical spread of the depression, in such a way that the spread is limited to the striatum only in the R192Q mutations, but more diffused to involve the hippocampus and the thalamus in the S218L mutation [200]. Thus, being highly susceptible to CSD, FHM1 patients develop more severe and prolonged hemiplegic auras. Motor deficits were significantly more prolonged (around 20 more minutes) in these FHM1 mutant mice when compared to the wild type [199]. 

### 5.2. FHM2

The gene responsible for the familial hemiplegic migraine type 2 (FHM2) was first identified in 2003 when the gene encoding the α2 subunit, the Na+/K+ ATPase, in neurons and astrocytes—ATP1A2 gene of chromosome 1q23—was discovered in two Italian families [201]. In essence, four α subunits have been identified for the Na+/K+ ATPase [202,203], with the testis-specific α4 subunit and the ubiquitous α1 subunit expressing no pathological mutations. However, the neuron-specific α3 subunit and the astrocyte-specific α2 subunit demonstrate mutations that cause neurological manifestations, essentially rapid-onset dystonia Parkinsonism and FHM2, respectively [204,205]. A more recent case study featuring a male adolescent who was diagnosed with familial hemiplegic migraine (FHM2) revealed a heterozygous genetic mutation within the ATP1A2 gene (c.1133C > T); this missense mutation may inhibit the function of the α2 subunit of the Na+/K+ ATPase [206].

#### 5.2.1. Na+/K+ ATPase

Na+/K+ ATPase pumps are essential for maintaining the resting membrane potential in neurons [207] and generating an ion gradient that is needed for neurotransmitter and nutrient uptake by the cells. As for the glial- and neuron-specific Na+/K+ ATPase pumps, they play an important role in clearing K+ ions from the synaptic cleft after neuronal transmission, a clearance that follows an initial fast phase and a late slow phase by driving K+ ions into the cells, while extruding Na+ ions to the outside [208,209]. This process is essential for the reuptake of glutamate from the synaptic cleft, which is mostly performed via the Na+-dependent glutamate uptake transporters primarily expressed in astrocytes [210,211]. Also, an actual physical association has been suggested linking this Na+/K+ ATPase subunit to glutamate transporters [212], and this was further asserted by an approximately identical localization of the α2 subunit of this Na+/K+ ATPase and glutamate transporters GLAST and GLUT1 in the somatosensory cortex of rats [213]. Hence, it would be logical to assume that the FHM2 mutations should be loss-of-function mutations, keeping high glutamate and/or K+ levels in the synaptic cleft, which can increase the susceptibility to CSD. The involvement of both α2 and α3 subunits of the Na+/K+ ATPase pumps in CSD has been shown in hippocampal slices, where the administration of ouabain, an inhibitor of the Na+/K+ ATPase, at concentrations that have minimal effects on the α1 subunit, significantly reduced the induction threshold for CSD via y increasing the extracellular levels of K+ [214].

#### 5.2.2. Specific Mutations

Several different mutations have been implicated in the pathogenesis of the disease, most of them being missense mutations [215,216,217,218]. Two specific mutations, W887R and L764P, have been shown to cause a loss of function in the Na+/K+ ATPase pumps, demonstrated by the inhibition of their currents while maintaining their plasma membrane expressions, suggesting the inactivation of these channels [219]. Other mutations, such as T345A, R689Q, and M731T, have normal function but altered kinetics, demonstrated by a decreased catalytic turnover and an increased affinity for extracellular K+ [220,221]. A study conducted by Leo et al. [222] generated knockin mice with the human W887R mutation responsible for FHM2. As expected, homozygous mutations were lethal. This was attributed to selective apoptosis in the amygdala and piriform cortex in response to the neuronal hyperactivity and to a depression of the brainstem reticular formation activity, demonstrated by an abolished respiration [223,224]. 

On the other hand, heterozygous mutations allowed for viable mice with a hypercontractile heart [225]. In essence, the study showed that, even though the mutant R887 allele is correctly transcribed and translated, it is sequestered by the endoplasmic reticulum and proteasome system, inhibiting its expression on the cell surface, in contrast to previous findings [222]. In vivo electrical cortical stimulation showed an increased susceptibility of the mutant mice to CSD when compared to the wild type, demonstrated through a decreased induction threshold and a higher propagation velocity [222]. This is most probably due to an accumulation of K+ in the synaptic cleft above physiological ranges, due to a decrease in the number and/or the activity of the α2 subunit of Na+/K+ ATPases in astrocytes, leading to a constant stimulation of the nervous system, eventually advancing to a CSD [181]. Other mutations were also noted in a large clinical investigation, comprising FHM2 patients alongside their clinical manifestations. Those with pure FHM had R65W, R202Q, R593W, and T762S variants in the ATP1A2 gene. Conversely, those with FHM and epilepsy displayed mutations such as R548C, E825K, and R928P in this gene. Individuals with FHM accompanied by epilepsy and intellectual disabilities harbored the T378N, G615R, and D718N mutants [226].

### 5.3. FHM3

Familial hemiplegic migraine type 3 (FHM3) was linked to a specific gene in 2005 after discovering a mutation in the SCN1A gene on chromosome 2q24 in three German families [227]. This gene encodes the α1 pore-forming subunit of the voltage-gated Na+ channel NaV1.1.

#### 5.3.1. Voltage-Gated Sodium Channels NaV1.1

The expression of NaV1.1 channels peaks during the third postnatal week, and then decreases dramatically to approximately half its peak expression in adult life. It is most likely localized to the brainstem, cortex, substantia nigra, and the caudate nucleus, as indicated by studies on adult rat brains [228]. These channels are mostly concentrated in the somatodendritic area, especially in hippocampal, pyramidal, and inhibitory neurons [229]. A study conducted by Yu et al. [230] showed that heterozygous and homozygous loss-of-function mutations of the SCN1A gene in Scn1a^+/−^ and Scn1a^−/−^ mice, respectively, experienced a decreased sodium current intensity in inhibitory GABAergic neurons, without any significant effect on excitatory neurons in the brain. Even though homozygous mice experienced ataxia and died on the 15th postnatal day, heterozygous mice suffered from seizures that led to severe myoclonic epilepsy in infancy (SMEI), and most were killed by the 21st postnatal day [230]. In essence, these findings suggest that the decreased sodium currents through mutant NaV1.1 channels in GABAergic neurons led to a decrease in GABA release throughout the nervous system. This phenomenon resulted in hyperexcitability responsible for the generation of seizures and epilepsies in affected mice. A study conducted by Gargus et al. [231] confirmed that the SCN1A gene known to be responsible for SMEI is, in fact, the exact gene responsible for the onset of FHM3. Thus, one would assume that a similar mechanism could also be found in FHM3 mutant NaV1.1 channels, where hyperexcitability could potentiate the appearance of CSD. 

#### 5.3.2. Specific Mutations

Even though a loss-of-function mutation was expected to be responsible for the pathogenesis of the migraine, as previously observed in the appearance of SMEI [230], FHM3 proved to result from gain-of-function mutations [232,233,234]. Jansen and colleagues [235] generated the first transgenic mouse model for FHM3 expressing the L263V mutation. The excessive firing of inhibitory GABAergic neurons could increase CSD susceptibility via increasing extracellular K+ concentrations [236]. In addition, Wiwanitkit [237] found that the FHM3 protein is more resistant than both FHM1 and FHM2.3.1. 

### 5.4. FHM4

Even though the involvement of three genes has been established in the onset of FHM, new research suggests the involvement of a fourth gene, PRRT2, in the rise of familial hemiplegic migraine. A novel case study featured a Portuguese patient with a heterozygous missense mutation (c.938C > T;p.Ala313Val), which inhibits the protein’s stability and subcellular localization [238]. In another study, a 13-year-old FHM patient who harbored a microdeletion in the chromosome 16p11.2 loci displayed a haploinsufficiency for the PRRT2 gene, which encodes a proline-rich transmembrane protein [239]. Further research studies are necessary to further elucidate the involvement of this gene in FHM; however, these physiological consequences indicate that the PRRT2 gene may be the fourth gene involved in the pathogenesis of FHM. 

#### 5.4.1. PRRT2 Protein

The PRRT2 protein is vital in proper neuronal development, healthy synaptic formation, and the release of neurotransmitters into the synaptic cleft. A variety of mutations in this gene, such as missense or deletions, has resulted in haploinsufficiency, which can be associated with various diseases, such as FHM or benign familial infantile epilepsy (BFIE) [240]. This protein is localized within the cortical layers of several neurological structures, such as the cerebral cortex, and may play a role in negatively modulating the Nav1.2 and Nav1.6 Na+ channels; mutations in this gene have led to hyperexcitability and an increased Na+ current in mutated neurons [241]. Thus, this protein is vital in maintaining neuronal network stability. A loss of function in this gene may be associated with synaptic deregulation or a decrease in the number of synapses, neuronal hyperexcitability, and the inhibition of the synchronous release of neurotransmitters by affected neurons [242]. 

#### 5.4.2. Specific Mutations

A genetic variant in the PRRT2 gene (NM_145239.3:c.938C > T; p.Ala313Val) was discovered via a WES family analysis in a 40-year-old male patient suffering from migraine with aura [243]. A physiological consequence of this missense mutation is disrupted protein stability; alterations in amino acid polarity impact the chemical dynamic between neighboring residues, which alters the three-dimensional folding of the protein. In another clinical study, twenty-two FHM patients from four families exhibited mutations in the PRRT2 gene as follows: c.649_650insC, c.649dupC, c.843C > G, and c.649dupC. Though limited, these studies indicate that mutations in the PRRT2 gene may be a genetic mechanism for hemiplegic migraine; however, further studies are needed to thoroughly examine the role of this gene [243].

## 6. Conclusions

It is crucial to study the history of migraine genetics and refer back to previously adapted techniques in its study. MA/MO genetics was studied initially using population and twin studies to learn about their heritability; then, many genetic techniques were used, including CGAS, GWAS, linkage studies, exome/genome sequencing, and RNA sequencing. Different loci were correlated to migraine using these techniques, with some of them pinned using more than one technique. Additionally, monogenic syndromes played a major role in identifying the genes responsible for migraine genetics. This review summarizes the major findings of the techniques used to study MO/MA genetics since its dawn. Additionally, great work has been completed to discern the genes responsible for FHM and SHM; we discussed the identified genes and their pathophysiological mechanisms which could be referred to for further reference. The study of migraine genetics has its limitations, including the diversity of techniques and results. Further studies are needed to advance this field further and decrease the ambiguities.

## Figures and Tables

**Table 2:** Pairwise concordance rate and proband-wise concordance rate in monozygotic (MZ) and dizygotic (DZ) twins, reported by [47,48,50,51,52]. (Inspired from Russel et al., 2001).

	Men		Women		Overall	
	MZ	DZ	MZ	DZ	MZ	DZ
Migraine with aura						
Pairwise concordance rate	36%	17%	32%	8%	34%	12%
Proband-wise concordance rate	53%	29%	48%	15%	50%	21%
Migraine without aura						
Pairwise concordance rate	17%	8%	33%	23%	28%	18%
Proband-wise concordance rate	29%	15%	50%	37%	43%	31%

**Table 3:** Summary of traditional linkage studies results (following the International Headache Classification (IHS) classification guidelines).

Articles	Country	Migraine Type	Genotyping Method	Chromosomal Locus
Nyholt et al., 2000 [37]	Australia	MA/MO	Regional microsatellite markers	Xq25-q28
Jones et al., 2001 [99]	USA	MA	Regional microsatellite markers	19p13
Carlsson et al., 2002 [96]	Sweden	MA/MO	Genome-wide scan	6p12.2-p21
Lea et al., 2002 [100]	Australia	MA/MO	Regional microsatellite markers	1q31
Wessman et al., 2002 [97]	Finland	MA	Genome-wide scan	4q24
Björnsson et al., 2003 [98]	Iceland	MO	Genome-wide scan	4q21
Cader, Noble-Topham et al., 2003 [101]	England	MA	Genome-wide scan	11q24
Soragna et al., 2003 [102]	Italy	MO	Genome-wide scan	14q21.2-q22.3
Russo et al., 2005 [95]	Italy	MA	Regional microsatellite markers	15q11-q13
Anttila et al., 2008 [103]	Australia and Finland	MA	Genome-wide scan	10q22-q23

**Table 4:** Summary of linkage studies performed with latent class analysis (LCA) and trait component analysis (TCA).

	Article	Country	Phenotypic Classification	Chromosome Locus
Latent Class Analysis				
	Nyholt et al., 2005/Anttila et al., 2006 [38,39]	Australia	Pulsation	5q21
	Anttila et al., 2006/Anttila et al., 2008 [39,103]	Australia and Finland	Migrainous headache	10q22-q23
	Anttila et al., 2008/Ligthart et al., 2008 [103,104]	Netherlands	Migrainous headache	10q22-q23
Trait Component Analysis				
	Nyholt et al., 2005/Anttila et al., 2006 [38,39]	Finland	Age at onset, photophobia, phonophobia, pain intensity, laterality, pulsation	4q24
	Anttila et al., 2006/Anttila et al., 2008 [39,103]	Finland	Pulsation	17p13
	Anttila et al., 2008/Ligthart et al., 2008 [103,104]	Australia and Finland	Laterality, pain intensity, phonophobia, photophobia, pulsation, nausea/vomiting	10q22-q23

**Table 5:** Summary of genome-wide association study (GWAS) results.

Article	Phenotype	Genes	Pathway
Anttila et al., 2010 [40]	MA	MTDH	Glutamate transport
		PGCP	Glutamate metabolism
Chasman et al., 2011 [108]	Migraine	TRPM8	Pain related
		LRP1	Neurotransmission
		PRDM16	Tissue structure and function [116]
Freilinger et al., 2012 [73]	MO	MEF2D	Neurotransmission
		ASTN2	TGF-beta signaling
		TGFBR2	TGF-beta signaling
		PHACTR1	Vascular endothelial function
Anttila et al., 2013 [107]	MA/MO	AJAP1	Metalloproteinase → tumor invasion
		TSPAN2	Metalloproteinase → tumor invasion
		FHL5	cAMP regulation
	MO	MMP6	Neurotransmission
		C7ORF10	Glutaric acid excretion
Gormley et al., 2016 [63]	MA/MO	SLC24A3	Ion homeostasis
		ITPK1	
		GJA1	
Gupta et al., 2016 [109] (phenome-wide AS)	Migraine	PHACTR1	Vascular endothelial function
Gerring et al., 2018 [111]	Migraine	NFKBIZ	Immune system and inflammation
		TNFSF10	
		TNFAIP3	
		CXCR4	
		ABCB1	
		NFIL3	
Guo et al., 2020 [109] (GWAS + transcriptome wide AS)	Migraine	ITGB5	Neurogenic inflammation, endothelial function, and calcium homeostasis
		SMG6	
		ADRA2B	
		ANKDD1B	
		KIAA0040	
Hautakangas et al., 2021 [112]	MA	HMOX2	Inflammation (vascular)
		CACNA1A	Voltage-dependent calcium channel (neurogenic)
		MPPED2	Metalloproteinase
	MO	SPINK2	Protease inhibitor
		FECH	Ferrochelatase

**Table 6:** Summary of studies using RNA sequencing and transcriptomic studies.

Articles	Phenotype	Genes	Pathways
Perry et al., 2016 [41] (Transcriptomic study)	Migraine	IL6	Inflammatory pathway
		SOCS3	
		IFNB	
		CXCR4	
		CCL2	
		NFKBIA	
Renthal et al., 2018 [122]	Migraine	CACNA1A	Ion channels
		SCN1A	
		NOTCH3	
Starobova et al., 2018 [123]	Pain	Neuropeptide Y	Ion channels
		SCN9A	
		SNC10A	
		SCN11A	
Jeong et al., 2018 [124]	Migraine	LRRC8	Immune response, glutamate signaling pathway, and reactive oxygen species regulation
		WSCD1	
Kogelman et al., 2019 [125]	MA	NMNAT2	Unknown
		RETN	
Vgontzas et al., 2020 [126]	MA, MO	HCK	Central Nervous System
		ARHGEF26	
		WSCD1	
		TSPAN2	
		NEGR1	
		SLC24A3	
		GPR182	Neurovascular cells
		NOTCH4	Peripheral Nervous System
		MYO1A	
		HELLS	
Kogelman et al., 2021 [127]	MA, MO	CPT1A	Fatty acid oxidation
		SLC25A20	
		ETFDH	Notch signaling pathways
		MAML2	
		ADAM15	
		ADAM17	
		CARD9	Immune-related pathways
		SH2D2A	
		CD300C	

**Table 7:** Summary of whole exome and whole genome sequencing studies (WES and WGS).

Article	Phenotype	Genes	Pathway
Williams et al., 2019 [42] (WES and WGS)	Migraine	ALPK1	Centrosome cilia functions
			Immune response and inflammation
Rasmussen et al., 2020 [129,130] (WGS and RNA seq)	MA/MO	ATXN1	Glutamate signaling
		FAM153B	Voltage-gated calcium channel
		CACNA1B	
Ibrahim et al., 2020 [128] (WES)	Migraine	ATP10A	ATPase
		ATP7B	
		CACNA1C	Voltage-gated calcium channel
		CACNA1I	

**Table 8:** ICHD criteria for familial hemiplegic migraine diagnosis [11].

A. Fulfilling hemiplegic migraine criteria	Attacks fulfilling the criteria for migraine with aura (Table 1). Aura consisting of both of the following: 2.1. fully reversible motor weakness, 2.2. fully reversible visual, sensory, and/or speech/language symptoms.
B. At least one first- or second-degree relative who experienced attacks fulfilling criteria in “A”.	

## Data Availability

No new data were gathered or analyzed. Data sharing is not applicable to this paper.

## Abbreviations

MA	Migraine with aura
MO	Migraine without aura
FHM	Familial hemiplegic migraine
CSD	Cortical spreading depression
ICHD-3	International Classification of Headache Disorders-3
CGAS	Candidate-gene association studies
LCA	Latent class analysis
TCA	Trait component analysis
GWAS	Genome-wide association study
MTHFR	Methylenetetrahydrofolate reductase

## References

[1] Headache Classification Committee of the International Headache Society (IHS) (2018). The International Classification of Headache Disorders, 3rd edition. Cephalalgia.

[2] de Boer I., Terwindt G.M., van den Maagdenberg A. (2020). Genetics of migraine aura: An update. J. Headache Pain.

[3] de Boer I., van den Maagdenberg A., Terwindt G.M. (2019). Advance in genetics of migraine. Curr. Opin. Neurol..

[4] Aguilar-Shea A.L., Membrilla Md J.A., Diaz-de-Teran J. (2022). Migraine review for general practice. Aten. Primaria.

[5] Steiner T.J., Stovner L.J., Jensen R., Uluduz D., Katsarava Z. (2020). Migraine remains second among the world’s causes of disability, and first among young women: Findings from GBD2019. J. Headache Pain.

[6] Sutherland H.G., Albury C.L., Griffiths L.R. (2019). Advances in genetics of migraine. J. Headache Pain.

[7] Hadjikhani N., Sanchez Del Rio M., Wu O., Schwartz D., Bakker D., Fischl B., Kwong K.K., Cutrer F.M., Rosen B.R., Tootell R.B.H. (2001). Mechanisms of migraine aura revealed by functional MRI in human visual cortex. Proc. Natl. Acad. Sci. USA.

[8] Bolay H., Reuter U., Dunn A.K., Huang Z., Boas D.A., Moskowitz M.A. (2002). Intrinsic brain activity triggers trigeminal meningeal afferents in a migraine model. Nat. Med..

[9] Zhang X., Levy D., Noseda R., Kainz V., Jakubowski M., Burstein R. (2010). Activation of meningeal nociceptors by cortical spreading depression: Implications for migraine with aura. J. Neurosci..

[10] Le H., Tfelt-Hansen P., Skytthe A., Kyvik K.O., Olesen J. (2012). Increase in self-reported migraine prevalence in the Danish adult population: A prospective longitudinal population-based study. BMJ Open.

[11] Headache Classification Committee of the International Headache Society (IHS) (2013). The international classification of headache disorders, (beta version). Cephalalgia.

[12] Schwedt T.J., Zhou J., Dodick D.W. (2014). Sporadic hemiplegic migraine with permanent neurological deficits. Headache J. Head. Face Pain.

[13] Riant F., Ducros A., Ploton C., Barbance C., Depienne C., Tournier-Lasserve E. (2010). De novo mutations in ATP1A2 and CACNA1A are frequent in early-onset sporadic hemiplegic migraine. Neurology.

[14] Black D.F. (2004). Sporadic hemiplegic migraine. Curr. Pain Headache Rep..

[15] Martínez E., Moreno R., López-Mesonero L., Vidriales I., Ruiz M., Guerrero A., Tellería J.J. (2016). Familial hemiplegic migraine with severe attacks: A new report with ATP1A2 mutation. Case Rep. Neurol. Med..

[16] Carreño O., Corominas R., Serra S.A., Sintas C., Fernández-Castillo N., Vila-Pueyo M., Toma C., Gené G.G., Pons R., Llaneza M. (2013). Screening of *CACNA1A* and *ATP1A2* genes in hemiplegic migraine: Clinical, genetic, and functional studies. Mol. Genet. Genom. Med..

[17] Di Cristofori A., Fusi L., Gomitoni A., Grampa G., Bersano A. (2012). R583Q CACNA1A variant in SHM1 and ataxia: Case report and literature update. J. Headache Pain.

[18] De Vries B., Freilinger T., Vanmolkot K.R.J., Koenderink J.B., Stam A.H., Terwindt G.M., Babini E., Van Den Boogerd E.H., Van Den Heuvel J.J.M.W., Frants R.R. (2007). Systematic analysis of three FHM genes in 39 sporadic patients with hemiplegic migraine. Neurology.

[19] Zangaladze A., Asadi-Pooya A.A., Ashkenazi A., Sperling M.R. (2010). Sporadic hemiplegic migraine and epilepsy associated with CACNA1A gene mutation. Epilepsy Behav..

[20] Jokubaitis M., Lengvenis G., Burnytė B., Audronytė E., Ryliškienė K. (2024). Case report: Late onset type 3 hemiplegic migraine with permanent neurologic sequelae after attacks. Front. Neurol..

[21] Ophoff R.A., Terwindt G.M., Vergouwe M.N., Van Eijk R., Oefner P.J., Hoffman S.M., Lamerdin J.E., Mohrenweiser H.W., Bulman D.E., Ferrari M. (1996). Familial hemiplegic migraine and episodic ataxia type-2 are caused by mutations in the Ca2+ channel gene CACNL1A4. Cell.

[22] Russell M.B., Ducros A. (2011). Sporadic and familial hemiplegic migraine: Pathophysiological mechanisms, clinical characteristics, diagnosis, and management. Lancet Neurol..

[23] Di Stefano V., Rispoli M.G., Pellegrino N., Graziosi A., Rotondo E., Napoli C., Pietrobon D., Brighina F., Parisi P. (2020). Diagnostic and therapeutic aspects of hemiplegic migraine. J. Neurol. Neurosurg. Psychiatry.

[24] Rasmussen B.K., Olesen J. (1992). Migraine with aura and migraine without aura: An epidemiological study. Cephalalgia.

[25] Ziegler D.K., Hur Y.-M., Bouchard T.J., Hassanein R.S., Barter R. (1998). Migraine in Twins Raised Together and Apart. Headache J. Head. Face Pain.

[26] Frosst P., Blom H.J., Milos R., Goyette P., Sheppard C.A., Matthews R.G., Boers G.J.H., Den Heijer M., Kluijtmans L.A.J., Van Den Heuve L.P. (1995). A candidate genetic risk factor for vascular disease: A common mutation in methylenetetrahydrofolate reductase. Nat. Genet..

[27] Kowa H., Yasui K., Takeshima T., Urakami K., Sakai F., Nakashima K. (2000). The homozygous C677T mutation in the methylenetetrahydrofolate reductase gene is a genetic risk factor for migraine. Am. J. Med. Genet..

[28] Kara I., Sazci A., Ergul E., Kaya G., Kilic G. (2003). Association of the C677T and A1298C polymorphisms in the 5,10 methylenetetrahydrofolate reductase gene in patients with migraine risk. Brain Res. Mol. Brain Res..

[29] Lea R.A., Ovcaric M., Sundholm J., Macmillan J., Griffiths L.R. (2004). The methylenetetrahydrofolate reductase gene variant C677T influences susceptibility to migraine with aura. BMC Med..

[30] Oterino A., Valle N., Bravo Y., Muñoz P., Sánchez-Velasco P., Ruiz-Alegría C., Castillo J., Leyva-Cobián F., Vadillo A., Pascual J. (2004). MTHFR T677 Homozygosis Influences the Presence of Aura in Migraineurs. Cephalalgia.

[31] Oterino A., Valle N., Pascual J., Bravo Y., Munoz P., Castillo J., Ruiz-Alegria C., Sanchez-Velasco P., Leyva-Cobian F., Cid C. (2005). Thymidylate synthase promoter tandem repeat and MTHFD1 R653Q polymorphisms modulate the risk for migraine conferred by the MTHFR T677 allele. Brain Res. Mol. Brain Res..

[32] Scher A.I., Terwindt G.M., Verschuren W.M.M., Kruit M.C., Blom H.J., Kowa H., Frants R.R., Van Den Maagdenberg A.M.J.M., Van Buchem M., Ferrari M.D. (2006). Migraine and MTHFR C677T genotype in a population-based sample. Ann. Neurol..

[33] Rubino E., Ferrero M., Rainero I., Binello E., Vaula G., Pinessi L. (2009). Association of the C677T polymorphism in the MTHFR gene with migraine: A meta-analysis. Cephalalgia.

[34] Liu R., Geng P., Ma M., Yu S., Yang M., He M., Dong Z., Zhang W. (2014). MTHFR C677T polymorphism and migraine risk: A meta-analysis. J. Neurol. Sci..

[35] Schürks M., Rist P.M., Kurth T. (2010). *MTHFR*677 C&gt;T and *ACE* D/I Polymorphisms in Migraine: A Systematic Review and Meta-Analysis. Headache J. Head. Face Pain.

[36] Samaan Z., Gaysina D., Cohen-Woods S., Craddock N., Jones L., Korszun A., Owen M., Mente A., McGuffin P., Farmer A. (2011). Methylenetetrahydrofolate Reductase Gene Variant (MTHFR C677T) and Migraine: A Case Control Study and Meta-analysis. BMC Neurol..

[37] Nyholt D.R., Curtain R.P., Griffiths L.R. (2000). Familial typical migraine: Significant linkage and localization of a gene to Xq24–28. Hum. Genet..

[38] Nyholt D.R., Morley K.I., Ferreira M.A.R., Medland S.E., Boomsma D.I., Heath A.C., Merikangas K.R., Montgomery G.W., Martin N.G. (2005). Genomewide Significant Linkage to Migrainous Headache on Chromosome 5q21. Am. J. Hum. Genet..

[39] Anttila V., Kallela M., Oswell G., Kaunisto M.A., Nyholt D.R., Hämäläinen E., Havanka H., Ilmavirta M., Terwilliger J., Sobel E. (2006). Trait Components Provide Tools to Dissect the Genetic Susceptibility of Migraine. Am. J. Hum. Genet..

[40] Anttila V. (2010). Genome-wide association study of migraine implicates a common susceptibility variant on 8q22.1. Nat. Genet..

[41] Perry C.J., Blake P., Buettner C., Papavassiliou E., Schain A.J., Bhasin M.K., Burstein R. (2016). Upregulation of inflammatory gene transcripts in periosteum of chronic migraineurs: Implications for extracranial origin of headache. Ann. Neurol..

[42] Williams L.B., Javed A., Sabri A., Morgan D.J., Huff C.D., Grigg J.R., Heng X.T., Khng A.J., Hollink I.H., Morrison M.A. (2019). ALPK1 missense pathogenic variant in five families leads to ROSAH syndrome, an ocular multisystem autosomal dominant disorder. Genet. Med..

[43] Russell M.B., Olesen J. (1993). The genetics of migraine without aura and migraine with aura. Cephalalgia.

[44] Russell M.B., Olesen J. (1995). Increased familial risk and evidence of genetic factor in migraine. BMJ.

[45] Russell M., Iselius L., Olesen J. (1995). Inheritance of migraine investigated by complex segregation analysis. Hum. Genet..

[46] Stewart W.F., Staffa J., Lipton R.B., Ottman R. (1997). Familial risk of migraine: A population-based study. Ann. Neurol..

[47] Ulrich V. (1999). The inheritance of migraine with aura estimated by means of structural equation modelling. J. Med. Genet..

[48] Ulrich V., Gervil M., Kyvik K.O., Olesen J., Russell M.B. (1999). Evidence of a genetic factor in migraine with aura: A population-based Danish twin study. Ann. Neurol..

[49] Waters W.E., O’Connor P.J. (1975). Prevalence of migraine. J. Neurol. Neurosurg. Psychiatry.

[50] Gervil M. (1999). Migraine without Aura: A Population-Based Twin Study. Am. Neurol. Assoc..

[51] Gervil M. (1999). The relative role of genetic and environmental factors in migraine without aura. Am. Acad. Neurol..

[52] Russell M.B. (2001). Genetics of migraine without aura, migraine with aura, migrainous disorder, head trauma migraine without aura and tension-type headache. Cephalalgia.

[53] Mulder E.J., Van Baal C., Gaist D., Kallela M., Kaprio J., Svensson D.A., Nyholt D.R., Martin N.G., Macgregor A.J., Cherkas L.F. (2003). Genetic and Environmental Influences on Migraine: A Twin Study Across Six Countries. Twin Res..

[54] Polderman T.J.C., Benyamin B., De Leeuw C.A., Sullivan P.F., Van Bochoven A., Visscher P.M., Posthuma D. (2015). Meta-analysis of the heritability of human traits based on fifty years of twin studies. Nat. Genet..

[55] Bigal M.E., Lipton R.B., Winner P., Reed M.L., Diamond S., Stewart W.F. (2007). Migraine in adolescents: Association with socioeconomic status and family history. Neurology.

[56] Russell M.B., Iselius L., Olesen J. (1996). Migraine without aura and migraine with aura are inherited disorders. Cephalalgia.

[57] Stewart W.F., Bigal M.E., Kolodner K., Dowson A., Liberman J.N., Lipton R.B. (2006). Familial risk of migraine Variation by proband age at onset and headache severity. Am. Acad. Neurol..

[58] Russell M.B., Ulrich V., Gervil M., Olesen J. (2002). Migraine Without Aura and Migraine With Aura Are Distinct Disorders. A Population-Based Twin Survey. Headache J. Head. Face Pain.

[59] Devoto M., Lozito A., Staffa G., D’Alessandro R., Sacquegna T., Romeo G. (1986). Segregation analysis of migraine in 128 families. Cephalalgia.

[60] Mochi M., Sangiorgi S., Cortelli P., Carelli V., Scapoli C., Crisci M., Monari L., Pierangeli G., Montagna P. (1993). Testing models for genetic determination of migraine. Cephalalgia.

[61] Montagna P. (2008). Migraine genetics. Expert Rev. Neurother..

[62] D’Amico D., Leone M., Macciardi F., Valentini S., Bussone G. (1991). Genetic transmission of migraine without aura: A study of 68 families. Ital. J. Neurol. Sci..

[63] Gormley P., Anttila V., Winsvold B.S., Palta P., Esko T., Pers T.H., Farh K.-H., Cuenca-Leon E., Muona M., Furlotte N.A. (2016). Meta-analysis of 375,000 individuals identifies 38 susceptibility loci for migraine. Nat. Genet..

[64] Nyholt D.R., Gillespie N.G., Heath A.C., Merikangas K.R., Duffy D.L., Martin N.G. (2004). Latent class and genetic analysis does not support migraine with aura and migraine without aura as separate entities. Genet. Epidemiol..

[65] Ligthart L., Boomsma D.I., Martin N.G., Stubbe J.H., Nyholt D.R. (2006). Migraine With Aura and Migraine Without Aura Are Not Distinct Entities: Further Evidence From a Large Dutch Population Study. Twin Res. Hum. Genet..

[66] de Vries B., Frants R.R., Ferrari M.D., van den Maagdenberg A.M. (2009). Molecular genetics of migraine. Hum. Genet..

[67] Launer L.J., Terwindt G.M., Ferrari M.D. (1999). The prevalence and characteristics of migraine in a population-based cohort: The GEM study. Neurology.

[68] Nyholt D.R., Borsook D., Griffiths L.R. (2017). Migrainomics—Identifying brain and genetic markers of migraine. Nat. Rev. Neurol..

[69] Gasparini C., Sutherland H., Griffiths L. (2013). Studies on the Pathophysiology and Genetic Basis of Migraine. Curr. Genom..

[70] Anttila V., Bulik-Sullivan B., Finucane H.K., Walters R.K., Bras J., Duncan L., Escott-Price V., Falcone G.J., Gormley P., Malik R. (2018). Analysis of shared heritability in common disorders of the brain. Science.

[71] Nyholt D.R., Anttila V., Winsvold B.S., Kurth T., Stefansson H., Kallela M., Malik R., Vries B.D., Terwindt G.M., Ikram M.A. (2015). Concordance of genetic risk across migraine subgroups: Impact on current and future genetic association studies. Cephalalgia.

[72] Zhao H., Eising E., De Vries B., Vijfhuizen L.S., Anttila V., Winsvold B.S., Kurth T., Stefansson H., Kallela M., Malik R. (2016). Gene-based pleiotropy across migraine with aura and migraine without aura patient groups. Cephalalgia.

[73] Freilinger T., Anttila V., De Vries B., Malik R., Kallela M., Terwindt G.M., Pozo-Rosich P., Winsvold B., Nyholt D.R., Van Oosterhout W.P.J. (2012). Genome-wide association analysis identifies susceptibility loci for migraine without aura. Nat. Genet..

[74] Dias A., Mariz T., Sousa A., Lemos C., Alves-Ferreira M. (2022). A review of migraine genetics: Gathering genomic and transcriptomic factors. Hum. Genet..

[75] Sepulveda-Sanchez J., Matia-Frances R., Martinez-Salio A., González-de la Aleja-Tejera J., Porta-Etessam J. (2004). Homocysteine and cerebrovascular disease. Rev. Neurol..

[76] Rozen R. (1997). Genetic predisposition to hyperhomocysteinemia: Deficiency of methylenetetrahydrofolate reductase (MTHFR). Thromb. Haemost..

[77] Todt U., Freudenberg J., Goebel I., Netzer C., Heinze A., Heinze-Kuhn K., Göbel H., Kubisch C. (2006). *MTHFR* C677T polymorphism and migraine with aura. Ann. Neurol..

[78] De Vries B., Anttila V., Freilinger T., Wessman M., Kaunisto M.A., Kallela M., Artto V., Vijfhuizen L.S., Göbel H., Dichgans M. (2016). Systematic re-evaluation of genes from candidate gene association studies in migraine using a large genome-wide association data set. Cephalalgia.

[79] Akerman S., Goadsby P. (2007). Dopamine and migraine: Biology and clinical implications. Cephalalgia.

[80] Chen J., Qin Z., Szele F., Bai G., Weiss B. (1991). Neuronal localization and modulation of the D2 dopamine receptor mRNA in brain of normal mice and mice lesioned with 6-hydroxydopamine. Neuropharmacology.

[81] Lazarov N., Pilgrim C. (1997). Localization of D1 and D2 dopamine receptors in the rat mesencephalic trigeminal nucleus by immunocytochemistry and in situ hybridization. Neurosci. Lett..

[82] Peterfreund R.A., Kosofsky B.E., Fink J.S. (1995). Cellular localization of dopamine D2 receptor messenger RNA in the rat trigeminal ganglion. Anesth. Analg..

[83] Bes A., Dupui P., Güell A., Bessoles G., Geraud G. (1986). Pharmacological exploration of dopamine hypersensitivity in migraine patients. Int. J. Clin. Pharmacol. Res..

[84] Piccini P., Pavese N., Palombo C., Pittella G., Distante A., Bonuccelli U. (1995). Transcranial Doppler ultrasound in migraine and tension-type headache after apomorphine administration: Double-blind crossover versus placebo study. Cephalalgia.

[85] Edvinsson L., Hardebo J., McCulloch J., Owman C. (1978). Effects of dopaminergic agonists and antagonists on isolated cerebral blood vessels. Acta Physiol. Scand..

[86] Erdal M.E., Herken H., Yilmaz M., Bayazit Y.A. (2001). Significance of the catechol-O-methyltransferase gene polymorphism in migraine. Mol. Brain Res..

[87] Gürsoy S., Erdal E., Herken H., Madenci E., Alaşehirli B., Erdal N. (2003). Significance of catechol-O-methyltransferase gene polymorphism in fibromyalgia syndrome. Rheumatol. Int..

[88] Hagen K., Pettersen E., Stovner L.J., Skorpen F., Zwart J.-A. (2006). The association between headache and Val158Met polymorphism in the catechol–O–methyltransferase gene: The HUNT Study. J. Headache Pain.

[89] Fernandez F., Colson N., Quinlan S., MacMillan J., Lea R., Griffiths L. (2009). Association between migraine and a functional polymorphism at the dopamine β-hydroxylase locus. Neurogenetics.

[90] Nyholt D.R., LaForge K.S., Kallela M., Alakurtti K., Anttila V., Farkkila M., Hamalainen E., Kaprio J., Kaunisto M.A., Heath A.C. (2008). A high-density association screen of 155 ion transport genes for involvement with common migraine. Hum. Mol. Genet..

[91] Curtain R., Tajouri L., Lea R., MacMillan J., Griffiths L. (2006). No mutations detected in the INSR gene in a chromosome 19p13 linked migraine pedigree. Eur. J. Med. Genet..

[92] McCarthy L.C., Hosford D.A., Riley J.H., Bird M.I., White N.J., Hewett D.R., Peroutka S.J., Griffiths L.R., Boyd P.R., Lea R.A. (2001). Single-nucleotide polymorphism alleles in the insulin receptor gene are associated with typical migraine. Genomics.

[93] Curtain R., Lea R.A., Quinlan S., Bellis C., Tajouri L., Hughes R., Macmillan J., Griffiths L.R. (2004). Investigation of the low-density lipoprotein receptor gene and cholesterol as a risk factor for migraine. J. Neurol. Sci..

[94] Mochi M., Cevoli S., Cortelli P., Pierangeli G., Scapoli C., Soriani S., Montagna P. (2003). Investigation of an LDLR gene polymorphism (19p13.2) in susceptibility to migraine without aura. J. Neurol. Sci..

[95] Russo L., Mariotti P., Sangiorgi E., Giordano T., Ricci I., Lupi F., Chiera R., Guzzetta F., Neri G., Gurrieri F. (2005). A New Susceptibility Locus for Migraine with Aura in the 15q11-q13 Genomic Region Containing Three GABA-A Receptor Genes. Am. J. Hum. Genet..

[96] Carlsson A., Forsgren L., Nylander P.-O., Hellman U., Forsman-Semb K., Holmgren G., Holmberg D., Holmberg M. (2002). Identification of a susceptibility locus for migraine with and without aura on 6p12.2-p21.1. Neurology.

[97] Wessman M., Kallela M., Kaunisto M.A., Marttila P., Sobel E., Hartiala J., Oswell G., Leal S.M., Papp J.C., Hämäläinen E. (2002). A Susceptibility Locus for Migraine with Aura, on Chromosome 4q24. Am. J. Hum. Genet..

[98] Björnsson Á., Gudmundsson G., Gudfinnsson E., Hrafnsdóttir M., Benedikz J., Skúladóttir S., Kristjánsson K., Frigge M.L., Kong A., Stefánsson K. (2003). Localization of a Gene for Migraine without Aura to Chromosome 4q21. Am. J. Hum. Genet..

[99] Jones K.W., Ehm M.G., Pericak-Vance M.A., Haines J.L., Boyd P.R., Peroutka S.J. (2001). Migraine with aura susceptibility locus on chromosome 19p13 is distinct from the familial hemiplegic migraine locus. Genomics.

[100] Lea R.A., Shepherd G.A., Curtain R.P., Nyholt D.R., Quinlan S., Brimage P.J., Griffiths L.R. (2002). A typical migraine susceptibility region localizes to chromosome 1q31. Neurogenetics.

[101] Cader Z.M., Noble-Topham S., Dyment D.A., Cherny S.S., Brown J.D., Rice G.P., Ebers G.C. (2003). Significant linkage to migraine with aura on chromosome 11q24. Hum. Mol. Genet..

[102] Soragna D., Vettori A., Carraro G., Marchioni E., Vazza G., Bellini S., Tupler R., Savoldi F., Mostacciuolo M. (2003). A locus for migraine without aura maps on chromosome 14q21.2-q22.3. Am. J. Hum. Genet..

[103] Anttila V., Nyholt D.R., Kallela M., Artto V., Vepsäläinen S., Jakkula E., Wennerström A., Tikka-Kleemola P., Kaunisto M.A., Hämäläinen E. (2008). Consistently Replicating Locus Linked to Migraine on 10q22-q23. Am. J. Hum. Genet..

[104] Ligthart L., Nyholt D.R., Hottenga J.-J., Distel M.A., Willemsen G., Boomsma D.I. (2008). A genome-wide linkage scan provides evidence for both new and previously reported loci influencing common migraine. Am. J. Med. Genet. Part. B Neuropsychiatr. Genet..

[105] Aulchenko Y.S., Hoppenbrouwers I.A., Ramagopalan S.V., Broer L., Jafari N., Hillert J., Link J., Lundström W., Greiner E., Dessa Sadovnick A. (2008). Genetic variation in the KIF1B locus influences susceptibility to multiple sclerosis. Nat. Genet..

[106] Hindorff L.A., Sethupathy P., Junkins H.A., Ramos E.M., Mehta J.P., Collins F.S., Manolio T.A. (2009). Potential etiologic and functional implications of genome-wide association loci for human diseases and traits. Proc. Natl. Acad. Sci. USA.

[107] Anttila V., Winsvold B.S., Gormley P., Kurth T., Bettella F., McMahon G., Kallela M., Malik R., De Vries B., Terwindt G. (2013). Genome-wide meta-analysis identifies new susceptibility loci for migraine. Nat. Genet..

[108] Chasman D.I., Schürks M., Anttila V., De Vries B., Schminke U., Launer L.J., Terwindt G.M., Van Den Maagdenberg A.M.J.M., Fendrich K., Völzke H. (2011). Genome-wide association study reveals three susceptibility loci for common migraine in the general population. Nat. Genet..

[109] Gupta R.M., Hadaya J., Trehan A., Zekavat S.M., Roselli C., Klarin D., Emdin C.A., Hilvering C.R., Bianchi V., Mueller C. (2017). A genetic variant associated with five vascular diseases is a distal regulator of endothelin-1 gene expression. Cell.

[110] Guo Y., Rist P.M., Daghlas I., Giulianini F., Kurth T., Chasman D.I. (2020). A genome-wide cross-phenotype meta-analysis of the association of blood pressure with migraine. Nat. Commun..

[111] Gerring Z.F., Powell J.E., Montgomery G.W., Nyholt D.R. (2018). Genome-wide analysis of blood gene expression in migraine implicates immune-inflammatory pathways. Cephalalgia.

[112] Hautakangas H., Winsvold B.S., Ruotsalainen S.E., Bjornsdottir G., Harder A.V., Kogelman L.J., Thomas L.F., Noordam R., Benner C., Gormley P. (2021). Genome-wide analysis of 102,084 migraine cases identifies 123 risk loci and subtype-specific risk alleles. MedRxiv.

[113] Kang D.-c., Su Z.-z., Sarkar D., Emdad L., Volsky D.J., Fisher P.B. (2005). Cloning and characterization of HIV-1-inducible astrocyte elevated gene-1, AEG-1. Gene.

[114] Noch E., Khalili K. (2009). Molecular mechanisms of necrosis in glioblastoma: The role of glutamate excitotoxicity. Cancer Biol. Ther..

[115] Iljazi A., Ayata C., Ashina M., Hougaard A. (2018). The role of endothelin in the pathophysiology of migraine—A systematic review. Curr. Pain Headache Rep..

[116] Ishibashi J., Seale P. (2015). Functions of Prdm16 in thermogenic fat cells. Temperature.

[117] Thimraj T.A., George L., Asrafuzzaman S., Upadhyay S., Ganguly K. (2018). Oxidative signaling in chronic obstructive airway diseases. Immunity and Inflammation in Health and Disease.

[118] Wong-Spracklen V.M., Kolesnik A., Eck J., Sabanathan S., Spasic-Boskovic O., Maw A., Baker K. (2022). Biallelic CACNA1A variants: Review of literature and report of a child with drug-resistant epilepsy and developmental delay. Am. J. Med. Genet. Part. A.

[119] Pellecchia S., Sepe R., Federico A., Cuomo M., Credendino S.C., Pisapia P., Bellevicine C., Nicolau-Neto P., Severo Ramundo M., Crescenzi E. (2019). The metallophosphoesterase-domain-containing protein 2 (MPPED2) gene acts as tumor suppressor in breast cancer. Cancers.

[120] Titus A., Marappa-Ganeshan R. (2023). Physiology, Endothelin. StatPearls.

[121] Wang Z., Gerstein M., Snyder M. (2009). RNA-Seq: A revolutionary tool for transcriptomics. Nat. Rev. Genet..

[122] Renthal W. (2018). Localization of migraine susceptibility genes in human brain by single-cell RNA sequencing. Cephalalgia.

[123] Starobova H., Himaya S.W.A., Lewis R.J., Vetter I. (2018). Transcriptomics in pain research: Insights from new and old technologies. Mol. Omics.

[124] Jeong H., Moye L.S., Southey B.R., Hernandez A.G., Dripps I., Romanova E.V., Rubakhin S.S., Sweedler J.V., Pradhan A.A., Rodriguez-Zas S.L. (2018). Gene network dysregulation in the trigeminal ganglia and nucleus accumbens of a model of chronic migraine-associated hyperalgesia. Front. Syst. Neurosci..

[125] Kogelman L.J., Falkenberg K., Halldorsson G.H., Poulsen L.U., Worm J., Ingason A., Stefansson H., Stefansson K., Hansen T.F., Olesen J. (2019). Comparing migraine with and without aura to healthy controls using RNA sequencing. Cephalalgia.

[126] Vgontzas A., Renthal W. (2020). Migraine-associated gene expression in cell types of the central and peripheral nervous system. Cephalalgia.

[127] Kogelman L.J., Falkenberg K., Buil A., Erola P., Courraud J., Laursen S.S., Michoel T., Olesen J., Hansen T.F. (2021). Changes in the gene expression profile during spontaneous migraine attacks. Sci. Rep..

[128] Ibrahim O., Sutherland H.G., Maksemous N., Smith R., Haupt L.M., Griffiths L.R. (2020). Exploring neuronal vulnerability to head trauma using a whole exome approach. J. Neurotrauma.

[129] Rasmussen A.H., Olofsson I., Chalmer M.A., Olesen J., Hansen T.F. (2020). Higher burden of rare frameshift indels in genes related to synaptic transmission separate familial hemiplegic migraine from common types of migraine. J. Med. Genet..

[130] Rasmussen A.H., Kogelman L.J., Kristensen D.M., Chalmer M.A., Olesen J., Hansen T.F. (2020). Functional gene networks reveal distinct mechanisms segregating in migraine families. Brain.

[131] Rudkjobing L.A., Esserlind A.-L., Olesen J. (2012). Future possibilities in migraine genetics. J. Headache Pain.

[132] Vaz-Drago R., Custódio N., Carmo-Fonseca M. (2017). Deep intronic mutations and human disease. Hum. Genet..

[133] Royal P., Andres-Bilbe A., Prado P.Á., Verkest C., Wdziekonski B., Schaub S., Baron A., Lesage F., Gasull X., Levitz J. (2019). Migraine-associated TRESK mutations increase neuronal excitability through alternative translation initiation and inhibition of TREK. Neuron.

[134] Gazerani P. (2019). Current evidence on potential uses of MicroRNA biomarkers for migraine: From diagnosis to treatment. Mol. Diagn. Ther..

[135] Tafuri E., Santovito D., de Nardis V., Marcantonio P., Paganelli C., Affaitati G., Bucci M., Mezzetti A., Giamberardino M.A., Cipollone F. (2015). MicroRNA profiling in migraine without aura: Pilot study. Ann. Med..

[136] Joutel A., Vahedi K., Corpechot C., Troesch A., Chabriat H., Vayssière C., Cruaud C., Maciazek J., Weissenbach J., Bousser M.-G. (1997). Strong clustering and stereotyped nature of Notch3 mutations in CADASIL patients. Lancet.

[137] Tan R.Y.Y., Markus H.S. (2016). CADASIL: Migraine, encephalopathy, stroke and their inter-relationships. PLoS ONE.

[138] Liem M.K., Oberstein S.A.L., van der Grond J., Ferrari M.D., Haan J. (2010). CADASIL and migraine: A narrative review. Cephalalgia.

[139] Piper R.D., Lambert G.A., Duckworth J.W. (1991). Cortical blood flow changes during spreading depression in cats. Am. J. Physiol.-Heart Circ. Physiol..

[140] Tfelt-Hansen P., Thorbjørn Jensen L., Olesen J. (2008). Delayed hyperperfusion following migraine with a prolonged aphasic aura in a patient with CADASIL. Cephalalgia.

[141] Eikermann-Haerter K., Wang Y., Dilekoz E., Arboleda-Velasquez J., Artavanis-Tsakonas S., Joutel A., Moskowitz M., Ayata C. (2009). Increased Susceptibility to Cortical Spreading Depression in CADASIL Mutant Mice. https://www.ncbi.nlm.nih.gov/pmc/articles/PMC3058390/.

[142] Kors E.E., Vanmolkot K.R., Haan J., Frants R.R., van den Maagdenberg A.M., Ferrari M.D. (2004). Recent findings in headache genetics. Curr. Opin. Neurol..

[143] Romero J.M., Rojas-Serrano L.F. (2023). Current Evaluation of Intracerebral Hemorrhage. Radiol. Clin..

[144] Itoh Y., Yamada M., Hayakawa M., Otomo E., Miyatake T. (1993). Cerebral amyloid angiopathy: A significant cause of cerebellar as well as lobar cerebral hemorrhage in the elderly. J. Neurol. Sci..

[145] Koemans E.A., Voigt S., Rasing I., van Etten E.S., van Zwet E.W., van Walderveen M.A., Wermer M.J., Terwindt G.M. (2020). Migraine with aura as early disease marker in hereditary Dutch-type cerebral amyloid angiopathy. Stroke.

[146] Agostoni E., Rigamonti A. (2012). Migraine and small vessel diseases. Neurol. Sci..

[147] Plaisier E., Ronco P., Adam M.P., Feldman J., Mirzaa G.M., Pagon R.A., Wallace S.E., Bean L.J.H., Gripp K.W., Amemiya A. (1993–2024). COL4A1-Related Disorders. GeneReviews® [Internet].

[148] Gould D.B., Phalan F.C., Van Mil S.E., Sundberg J.P., Vahedi K., Massin P., Bousser M.G., Heutink P., Miner J.H., Tournier-Lasserve E. (2006). Role of COL4A1 in small-vessel disease and hemorrhagic stroke. N. Engl. J. Med..

[149] Lanfranconi S., Markus H.S. (2010). COL4A1 mutations as a monogenic cause of cerebral small vessel disease: A systematic review. Stroke.

[150] Xu Y., Padiath Q.S., Shapiro R.E., Jones C.R., Wu S.C., Saigoh N., Saigoh K., Ptáček L.J., Fu Y.-H. (2005). Functional consequences of a CKIδ mutation causing familial advanced sleep phase syndrome. Nature.

[151] Cheong J.K., Virshup D.M. (2011). Casein kinase 1: Complexity in the family. Int. J. Biochem. Cell Biol..

[152] Knippschild U., Gocht A., Wolff S., Huber N., Löhler J., Stöter M. (2005). The casein kinase 1 family: Participation in multiple cellular processes in eukaryotes. Cell. Signal..

[153] Toh K.L., Jones C.R., He Y., Eide E.J., Hinz W.A., Virshup D.M., Ptácek L.J., Fu Y.-H. (2001). An h Per2 phosphorylation site mutation in familial advanced sleep phase syndrome. Science.

[154] Lee H., Chen R., Lee Y., Yoo S., Lee C. (2009). Essential roles of CKIδ and CKIε in the mammalian circadian clock. Proc. Natl. Acad. Sci. USA.

[155] Brennan K., Bates E.A., Shapiro R.E., Zyuzin J., Hallows W.C., Huang Y., Lee H.-Y., Jones C.R., Fu Y.-H., Charles A.C. (2013). Casein kinase iδ mutations in familial migraine and advanced sleep phase. Sci. Transl. Med..

[156] Lafrenière R.G., Rouleau G.A. (2011). Migraine: Role of the TRESK two-pore potassium channel. Int. J. Biochem. Cell Biol..

[157] Kowalska M., Prendecki M., Kapelusiak-Pielok M., Grzelak T., Łagan-Jędrzejczyk U., Wiszniewska M., Kozubski W., Dorszewska J. (2020). Analysis of Genetic Variants in SCN1A, SCN2A, KCNK18, TRPA1 and STX1A as a Possible Marker of Migraine. Curr. Genom..

[158] Jen J., Cohen A., Yue Q., Stout J., Vinters H., Nelson S., Baloh R. (1997). Hereditary endotheliopathy with retinopathy, nephropathy, and stroke (HERNS). Neurology.

[159] Elliott D. (2008). Migraine and stroke: Current perspectives. Neurol. Res..

[160] Salloway S., Cummings J. (1994). Subcortical disease and neuropsychiatric illness. J. Neuropsychiatry Clin. Neurosci..

[161] Hirano M., Pavlakis S.G. (1994). Topical review: Mitochondrial myopathy, encephalopathy, lactic acidosis, and strokelike episodes (MELAS): Current concepts. J. Child. Neurol..

[162] Hirano M., Ricci E., Koenigsberger M.R., Defendini R., Pavlakis S.G., DeVivo D.C., DiMauro S., Rowland L.P. (1992). MELAS: An original case and clinical criteria for diagnosis. Neuromuscul. Disord..

[163] Ohno K., Isotani E., Hirakawa K. (1997). MELAS presenting as migraine complicated by stroke: Case report. Neuroradiology.

[164] Sproule D.M., Kaufmann P. (2008). Mitochondrial encephalopathy, lactic acidosis, and strokelike episodes: Basic concepts, clinical phenotype, and therapeutic management of MELAS syndrome. Ann. N. Y. Acad. Sci..

[165] Montagna P., Gallassi R., Medori R., Govoni E., Zeviani M., Di Mauro S., Lugaresi E., Andermann F. (1988). MELAS syndrome: Characteristic migrainous and epileptic features and maternal transmission. Neurology.

[166] Nakagawa M., Osame M. (1990). Clinical aspects of mitochondrial encephalomyopathy—Abnormality of mitochondrial respiratory chain. No Shinkei Brain Nerve.

[167] Wong L.J.C. (2007). Pathogenic mitochondrial DNA mutations in protein-coding genes. Muscle Nerve Off. J. Am. Assoc. Electrodiagn. Med..

[168] Buzzi M.G., Di Gennaro G., D’Onofrio M., Ciccarelli O., Santorelli F., Fortini D., Nappi G., Nicoletti F., Casali C. (2000). mtDNA A3243G MELAS mutation is not associated with multigenerational female migraine. Neurology.

[169] Cevoli S., Pallotti F., Morgia C.L., Valentino M.L., Pierangeli G., Cortelli P., Baruzzi A., Montagna P., Carelli V. (2010). High frequency of migraine-only patients negative for the 3243 A>G tRNALeu mtDNA mutation in two MELAS families. Cephalalgia.

[170] Wilms A., de Boer I., Terwindt G. (2022). Retinal Vasculopathy with Cerebral Leukoencephalopathy and Systemic manifestations (RVCL-S): An update on basic science and clinical perspectives. Cereb. Circ. Cogn. Behav..

[171] Ford A.L., Chin V.W., Fellah S., Binkley M.M., Bodin A.M., Balasetti V., Taiwo Y., Kang P., Lin D., Jen J.C. (2020). Lesion evolution and neurodegeneration in RVCL-S: A monogenic microvasculopathy. Neurology.

[172] Stam A.H., Kothari P.H., Shaikh A., Gschwendter A., Jen J.C., Hodgkinson S., Hardy T.A., Hayes M., Kempster P.A., Kotschet K.E. (2016). Retinal vasculopathy with cerebral leukoencephalopathy and systemic manifestations. Brain.

[173] Mateen F., Krecke K., Younge B., Ford A., Shaikh A., Kothari P., Atkinson J. (2010). Evolution of a tumor-like lesion in cerebroretinal vasculopathy and TREX1 mutation. Neurology.

[174] DiFrancesco J.C., Novara F., Zuffardi O., Forlino A., Gioia R., Cossu F., Bolognesi M., Andreoni S., Saracchi E., Frigeni B. (2015). TREX1 C-terminal frameshift mutations in the systemic variant of retinal vasculopathy with cerebral leukodystrophy. Neurol. Sci..

[175] Pelzer N., Hoogeveen E., Haan J., Bunnik R., Poot C., van Zwet E., Inderson A., Fogteloo A., Reinders M., Middelkoop H. (2019). Systemic features of retinal vasculopathy with cerebral leukoencephalopathy and systemic manifestations: A monogenic small vessel disease. J. Intern. Med..

[176] Simard J.M., Francisco G.-B., Ballinger W.E., Mickle J.P., Quisling R.G. (1986). Cavernous angioma: A review of 126 collected and 12 new clinical cases. Neurosurgery.

[177] Requena I., Arias M., Lopez-Ibor L., Pereiro I., Barba A., Alonso A., Monton E. (1991). Cavernomas of the central nervous system: Clinical and neuroimaging manifestations in 47 patients. J. Neurol. Neurosurg. Psychiatry.

[178] Chen D.-H., Lipe H.P., Qin Z., Bird T.D. (2002). Cerebral cavernous malformation: Novel mutation in a Chinese family and evidence for heterogeneity. J. Neurol. Sci..

[179] Lehnhardt F.-G., von Smekal U., Rückriem B., Stenzel W., Neveling M., Heiss W.-D., Jacobs A.H. (2005). Value of gradient-echo magnetic resonance imaging in the diagnosis of familial cerebral cavernous malformation. Arch. Neurol..

[180] Thomsen L.L., Eriksen M.K., Romer S.F., Andersen I., Ostergaard E., Keiding N., Olesen J., Russell M. (2002). An epidemiological survey of hemiplegic migraine. Cephalalgia.

[181] Pietrobon D. (2007). Familial hemiplegic migraine. Neurotherapeutics.

[182] Joutel A., Ducros A., Vahedi K., Labauge P., Delrieu O., Pinsard N., Mancini J., Ponsot G., Gouttiere F., Gastaut J. (1994). Genetic heterogeneity of familial hemiplegic migraine. Am. J. Hum. Genet..

[183] Thomsen L., Eriksen M., Roemer S., Andersen I., Olesen J., Russell M. (2002). A population-based study of familial hemiplegic migraine suggests revised diagnostic criteria. Brain.

[184] Ducros A., Denier C., Joutel A., Cecillon M., Lescoat C., Vahedi K., Darcel F., Vicaut E., Bousser M.-G., Tournier-Lasserve E. (2001). The clinical spectrum of familial hemiplegic migraine associated with mutations in a neuronal calcium channel. New Engl. J. Med..

[185] Ducros A., Joutel A., Cecillon M., Tournier-Lasserve E., Vahedi K., Bousser M.G., Ferreira A., Bernard E., Verier A., Echenne B. (1997). Mapping of a second locus for familial hemiplegic migraine to 1q21–q23 and evidence of further heterogeneity. Ann. Neurol. Off. J. Am. Neurol. Assoc. Child. Neurol. Soc..

[186] Hansen J.M., Hauge A.W., Ashina M., Olesen J. (2011). Trigger factors for familial hemiplegic migraine. Cephalalgia.

[187] Chabriat H., Vahedi K., Clark C., Poupon C., Ducros A., Denier C., Le Bihan D., Bousser M. (2000). Decreased hemispheric water mobility in hemiplegic migraine related to mutation of CACNA1A gene. Neurology.

[188] Deprez L., Weckhuysen S., Peeters K., Deconinck T., Claeys K.G., Claes L.R., Suls A., Van Dyck T., Palmini A., Matthijs G. (2008). Epilepsy as part of the phenotype associated with ATP1A2 mutations. Epilepsia.

[189] Pietrobon D. (2005). Function and dysfunction of synaptic calcium channels: Insights from mouse models. Curr. Opin. Neurobiol..

[190] van den Maagdenberg A.M., Frants R.R. (2005). Migraine genetics: An update. Curr. Pain Headache Rep..

[191] Dichgans M., Herzog J., Freilinger T., Wilke M., Auer D. (2005). 1H-MRS alterations in the cerebellum of patients with familial hemiplegic migraine type 1. Neurology.

[192] de Boer I., Hansen J.M., Terwindt G.M. (2024). Hemiplegic migraine. Handb. Clin. Neurol..

[193] Sanchez Del Rio M., Cutrer F.M. (2023). Pathophysiology of migraine aura. Handb. Clin. Neurol..

[194] Loonen I.C.M., Voskuyl R.A., Schenke M., van Heiningen S.H., van den Maagdenberg A., Tolner E.A. (2024). Spontaneous and optogenetically induced cortical spreading depolarization in familial hemiplegic migraine type 1 mutant mice. Neurobiol. Dis..

[195] Ayata C., Shimizu-Sasamata M., Lo E., Noebels J., Moskowitz M. (1999). Impaired neurotransmitter release and elevated threshold for cortical spreading depression in mice with mutations in the α1A subunit of P/Q type calcium channels. Neuroscience.

[196] Wakamori M., Yamazaki K., Matsunodaira H., Teramoto T., Tanaka I., Niidome T., Sawada K., Nishizawa Y., Sekiguchi N., Mori E. (1998). Single tottering mutations responsible for the neuropathic phenotype of the P-type calcium channel. J. Biol. Chem..

[197] van den Maagdenberg A.M., Pietrobon D., Pizzorusso T., Kaja S., Broos L.A., Cesetti T., van de Ven R.C., Tottene A., van der Kaa J., Plomp J.J. (2004). A Cacna1a knockin migraine mouse model with increased susceptibility to cortical spreading depression. Neuron.

[198] Tottene A., Conti R., Fabbro A., Vecchia D., Shapovalova M., Santello M., van den Maagdenberg A.M., Ferrari M.D., Pie-trobon D. (2009). Enhanced excitatory transmission at cortical synapses as the basis for facilitated spreading depression in Ca(v)2.1 knockin migraine mice. Neuron.

[199] Eikermann-Haerter K., Dileköz E., Kudo C., Savitz S.I., Waeber C., Baum M.J., Ferrari M.D., van den Maagdenberg A.M., Moskowitz M.A., Ayata C. (2009). Genetic and hormonal factors modulate spreading depression and transient hemiparesis in mouse models of familial hemiplegic migraine type 1. J. Clin. Investig..

[200] Eikermann-Haerter K., Yuzawa I., Qin T., Wang Y., Baek K., Kim Y.R., Hoffmann U., Dilekoz E., Waeber C., Ferrari M.D. (2011). Enhanced subcortical spreading depression in familial hemiplegic migraine type 1 mutant mice. J. Neurosci..

[201] De Fusco M., Marconi R., Silvestri L., Atorino L., Rampoldi L., Morgante L., Ballabio A., Aridon P., Casari G. (2003). Haploinsufficiency of ATP1A2 encoding the Na. Nat. Genet..

[202] Blanco G., Mercer R.W. (1998). Isozymes of the Na-K-ATPase: Heterogeneity in structure, diversity in function. Am. J. Physiol. Ren. Physiol..

[203] Crambert G., Hasler U., Beggah A.T., Yu C., Modyanov N.N., Horisberger J.-D., Lelievre L., Geering K.t. (2000). Transport and pharmacological properties of nine different human Na, K-ATPase isozymes. J. Biol. Chem..

[204] de Carvalho Aguiar P., Sweadner K.J., Penniston J.T., Zaremba J., Liu L., Caton M., Linazasoro G., Borg M., Tijssen M.A., Bressman S.B. (2004). Mutations in the Na+/K+-ATPase α3 gene ATP1A3 are associated with rapid-onset dystonia parkinsonism. Neuron.

[205] McGrail K., Phillips J., Sweadner K. (1991). Immunofluorescent localization of three Na, K-ATPase isozymes in the rat central nervous system: Both neurons and glia can express more than one Na, K-ATPase. J. Neurosci..

[206] Zhang H., Jiang L., Xian Y., Yang S. (2024). Familial hemiplegic migraine type 2: A case report of an adolescent with ATP1A2 mutation. Front. Neurol..

[207] Wright S.H. (2004). Generation of resting membrane potential. Adv. Physiol. Educ..

[208] D’Ambrosio R., Gordon D.S., Winn H.R. (2002). Differential role of KIR channel and Na+/K+-pump in the regulation of extracellular K+ in rat hippocampus. J. Neurophysiol..

[209] Ransom C.B., Ransom B.R., Sontheimer H. (2000). Activity-dependent extracellular K+ accumulation in rat optic nerve: The role of glial and axonal Na+ pumps. J. Physiol..

[210] Anderson C.M., Swanson R.A. (2000). Astrocyte glutamate transport: Review of properties, regulation, and physiological functions. Glia.

[211] Rose C.R., Ziemens D., Untiet V., Fahlke C. (2018). Molecular and cellular physiology of sodium-dependent glutamate transporters. Brain Res. Bull..

[212] Rose E.M., Koo J.C., Antflick J.E., Ahmed S.M., Angers S., Hampson D.R. (2009). Glutamate transporter coupling to Na, K-ATPase. J. Neurosci..

[213] Cholet N., Pellerin L., Magistretti P., Hamel E. (2002). Similar perisynaptic glial localization for the Na+, K+-ATPase α2 subunit and the glutamate transporters GLAST and GLT-1 in the rat somatosensory cortex. Cereb. Cortex.

[214] Haglund M.M., Schwartzkroin P.A. (1990). Role of Na-K pump potassium regulation and IPSPs in seizures and spreading depression in immature rabbit hippocampal slices. J. Neurophysiol..

[215] Hoefnagels W., Black D., Sandkuijl L., Frants R., Ferrari M., van den Maagdenberg A. (2003). Novel mutations in the Na?, K?-ATPase pump gene ATP1A2 associated with familial hemiplegic migraine and benign familial infantile convulsions. Ann. Neurol..

[216] Spadaro M., Ursu S., Lehmann-Horn F., Liana V., Giovanni A., Paola G., Frontali M., Jurkat-Rott K. (2004). A G301R Na+/K+-ATPase mutation causes familial hemiplegic migraine type 2 with cerebellar signs. Neurogenetics.

[217] Jurkat-Rott K., Freilinger T., Dreier J., Herzog J., Göbel H., Petzold G., Montagna P., Gasser T., Lehmann-Horn F., Dichgans M. (2004). Variability of familial hemiplegic migraine with novel A1A2 Na+/K+-ATPase variants. Neurology.

[218] Riant F., De Fusco M., Aridon P., Ducros A., Ploton C., Marchelli F., Maciazek J., Bousser M.G., Casari G., Tournier-Lasserve E. (2005). ATP1A2 mutations in 11 families with familial hemiplegic migraine. Hum. Mutat..

[219] Koenderink J.B., Zifarelli G., Qiu L.Y., Schwarz W., De Pont J.J.H., Bamberg E., Friedrich T. (2005). Na, K-ATPase mutations in familial hemiplegic migraine lead to functional inactivation. Biochim. Biophys. Acta (BBA)-Biomembr..

[220] Segall L., Mezzetti A., Scanzano R., Gargus J.J., Purisima E., Blostein R. (2005). Alterations in the α2 isoform of Na, K-ATPase associated with familial hemiplegic migraine type 2. Proc. Natl. Acad. Sci. USA.

[221] Vanmolkot K.R., Kors E.E., Hottenga J.J., Terwindt G.M., Haan J., Hoefnagels W.A., Black D.F., Sandkuijl L.A., Frants R.R., Ferrari M.D. (2003). Novel mutations in the Na+, K+-ATPase pump gene ATP1A2 associated with familial hemiplegic migraine and benign familial infantile convulsions. Ann. Neurol..

[222] Leo L., Gherardini L., Barone V., De Fusco M., Pietrobon D., Pizzorusso T., Casari G. (2011). Increased susceptibility to cortical spreading depression in the mouse model of familial hemiplegic migraine type 2. PLoS Genet..

[223] Ikeda K., Onaka T., Yamakado M., Nakai J., Ishikawa T.-o., Taketo M.M., Kawakami K. (2003). Degeneration of the amygdala/piriform cortex and enhanced fear/anxiety behaviors in sodium pump α2 subunit (Atp1a2)-deficient mice. J. Neurosci..

[224] Ikeda K., Onimaru H., Yamada J., Inoue K., Ueno S., Onaka T., Toyoda H., Arata A., Ishikawa T.-o., Taketo M.M. (2004). Malfunction of respiratory-related neuronal activity in Na+, K+-ATPase α2 subunit-deficient mice is attributable to abnormal Cl-homeostasis in brainstem neurons. J. Neurosci..

[225] James P.F., Grupp I.L., Grupp G., Woo A.L., Askew G.R., Croyle M.L., Walsh R.A., Lingrel J.B. (1999). Identification of a specific role for the Na, K-ATPase α2 isoform as a regulator of calcium in the heart. Mol. Cell.

[226] Li Y., Tang W., Kang L., Kong S., Dong Z., Zhao D., Liu R., Yu S. (2021). Functional correlation of ATP1A2 mutations with phenotypic spectrum: From pure hemiplegic migraine to its variant forms. J. Headache Pain.

[227] Dichgans M., Freilinger T., Eckstein G., Babini E., Lorenz-Depiereux B., Biskup S., Ferrari M.D., Herzog J., van den Maagdenberg A.M., Pusch M. (2005). Mutation in the neuronal voltage-gated sodium channel SCN1A in familial hemiplegic migraine. Lancet.

[228] Gong B., Rhodes K.J., Bekele-Arcuri Z., Trimmer J.S. (1999). Type I and type II Na+ channel α-subunit polypeptides exhibit distinct spatial and temporal patterning, and association with auxiliary subunits in rat brain. J. Comp. Neurol..

[229] Johnston D., Magee J.C., Colbert C.M., Christie B.R. (1996). Active properties of neuronal dendrites. Annu. Rev. Neurosci..

[230] Yu F.H., Mantegazza M., Westenbroek R.E., Robbins C.A., Kalume F., Burton K.A., Spain W.J., McKnight G.S., Scheuer T., Catterall W.A. (2006). Reduced sodium current in GABAergic interneurons in a mouse model of severe myoclonic epilepsy in infancy. Nat. Neurosci..

[231] Gargus J.J., Tournay A. (2007). Novel mutation confirms seizure locus SCN1A is also familial hemiplegic migraine locus FHM3. Pediatr. Neurol..

[232] Dhifallah S., Lancaster E., Merrill S., Leroudier N., Mantegazza M., Cestèle S. (2018). Gain of function for the SCN1A/hNav1. 1-L1670W mutation responsible for familial hemiplegic migraine. Front. Mol. Neurosci..

[233] Cestèle S., Schiavon E., Rusconi R., Franceschetti S., Mantegazza M. (2013). Nonfunctional NaV1. 1 familial hemiplegic migraine mutant transformed into gain of function by partial rescue of folding defects. Proc. Natl. Acad. Sci. USA.

[234] Kahlig K.M., Rhodes T.H., Pusch M., Freilinger T., Pereira-Monteiro J.M., Ferrari M.D., Van Den Maagdenberg A.M., Dichgans M., George A.L. (2008). Divergent sodium channel defects in familial hemiplegic migraine. Proc. Natl. Acad. Sci. USA.

[235] Jansen N.A., Dehghani A., Linssen M.M., Breukel C., Tolner E.A., van den Maagdenberg A.M. (2020). First FHM3 mouse model shows spontaneous cortical spreading depolarizations. Ann. Clin. Transl. Neurol..

[236] Desroches M., Faugeras O., Krupa M., Mantegazza M. (2019). Modeling cortical spreading depression induced by the hyperactivity of interneurons. J. Comput. Neurosci..

[237] Wiwanitkit V. (2009). FHM3 in familial hemiplegic migraine is more resistant to mutation than FHM1 and FHM2. J. Neurol. Sci..

[238] Dias A., Santos M., Carvalho E., Felicio D., Silva P., Alves I., Pinho T., Sousa A., Alves-Ferreira M., Lemos C. (2023). Functional characterization of a novel PRRT2 variant found in a Portuguese patient with hemiplegic migraine. Clin. Genet..

[239] Sen K., Genser I., DiFazio M., DiSabella M. (2022). Haploinsufficiency of PRRT2 Leading to Familial Hemiplegic Migraine in Chromosome 16p11.2 Deletion Syndrome. Neuropediatrics.

[240] Nandyala A., Shah T., Ailani J. (2023). Hemiplegic Migraine. Curr. Neurol. Neurosci. Rep..

[241] Suzuki-Muromoto S., Kosaki R., Kosaki K., Kubota M. (2020). Familial hemiplegic migraine with a PRRT2 mutation: Phenotypic variations and carbamazepine efficacy. Brain Dev..

[242] Méneret A., Gaudebout C., Riant F., Vidailhet M., Depienne C., Roze E. (2013). PRRT2 mutations and paroxysmal disorders. Eur. J. Neurol..

[243] Hasirci Bayir B.R., Tutkavul K., Eser M., Baykan B. (2021). Epilepsy in patients with familial hemiplegic migraine. Seizure.

